# Coverage of the influenza and pneumococcal vaccinations among immigrant and non-immigrant older adults in Canada: a cross-sectional analysis of data from the Canadian Longitudinal Study on Aging (CLSA)

**DOI:** 10.1186/s12889-025-25005-z

**Published:** 2025-11-04

**Authors:** Ji Yoon Kim, Giorgia Sulis, Alton Russell, Seungmi Yang, Jesse Papenburg, Ananya Banerjee, Patricia Li

**Affiliations:** 1 Department of Epidemiology, Biostatistics and Occupational Health, School of Population and Global Health, Faculty of Medicine and Health Sciences, McGill University, Montreal, QC Canada; 2 School of Epidemiology and Public Health, Faculty of Medicine, University of Ottawa, Ottawa, ON Canada; 3 Methodological and Implementation Research Program, Ottawa Hospital Research Institute, Ottawa, ON Canada; 4 Department of Pediatrics, Faculty of Medicine, Research Institute, McGill University, McGill University Health Centre, Montreal, QC Canada

**Keywords:** Vaccination coverage, Immigrant health, Vaccine equity, Health equity, Influenza, Pneumococcal disease, The Canadian Longitudinal Study on Aging (CLSA)

## Abstract

**Background:**

Influenza and pneumococcal vaccination coverage in older adults fall below the target of 80%. Being an immigrant may be associated with lower coverage of both vaccinations, but limited efforts have been made in the Canadian context to explore such disparities. Therefore, we examined the association between immigrant status and coverage of influenza and pneumococcal vaccinations among older adults as well as the relative importance of immigrant status in predicting coverage of both vaccinations.

**Methods:**

We conducted a cross-sectional secondary analysis of the Canadian Longitudinal Study on Aging data. We descriptively analyzed coverage of both vaccinations by immigrant status and used Poisson regression models with robust standard errors to estimate the associations of immigrant status and other key equity stratifiers with vaccination. Importance of various determinants, including immigrant status, in predicting both vaccinations were assessed using random forest algorithms.

**Results:**

Immigrant participants reported lower coverage of influenza vaccination in the past 12 months (63.8% [95% CI: 60.9–66.7%] vs. 66.9% [95% CI: 65.5–68.3%]) and pneumococcal vaccination ever (48.7% [95% CI: 45.6–51.8%] vs. 55.8% [95% CI: 54.3–57.3%]). Prevalence of influenza and pneumococcal vaccinations were both lower among immigrant participants compared to non-immigrant participants. Immigrant status was among the 10 most important predictors of pneumococcal vaccination, but among the less important predictors of influenza vaccination.

**Conclusions:**

Overall, we found disparities in influenza and pneumococcal vaccination by immigrant status among older adults in Canada. Further studies on vaccination coverage and decision-making among marginalized communities, including immigrants, are warranted to equitably improve vaccine uptake.

**Supplementary Information:**

The online version contains supplementary material available at 10.1186/s12889-025-25005-z.

## Background

Influenza and pneumonia rank among the top 10 leading causes of death in Canada [1]. Older age is a risk factor for the incidence and severity of influenza and pneumococcal disease [2]. Currently, in Canada, adults aged 65 years and older are recommended by the National Advisory Committee on Immunization (NACI) to receive an influenza vaccination every influenza season, particularly high-dose, adjuvanted, or recombinant influenza vaccines [3]. Those aged 65 years and older are also recommended to receive a dose of either the 20-valent or the 21-valent pneumococcal vaccine regardless of their previous pneumococcal vaccination history [4]. Notably, recommendations for influenza and pneumococcal vaccinations have changed over time. For instance, adults aged 65 years and older were recommended to receive a dose of the 23-valent pneumococcal vaccine since October 2014 and either the 20-valent or 21-valent pneumococcal vaccine since April 2025 [5]. Influenza and pneumococcal vaccinations are publicly funded in all jurisdictions in Canada for adults aged 65 years and older [6].

Despite these recommendations for and public funding of vaccinations, results from the 2023 Adult National Immunization Coverage Survey suggest that the national coverage of influenza and pneumococcal vaccines in adults aged 65 years and older was estimated to be 70.2% and 54.7%, respectively [7], well below the target of 80% [8]. Furthermore, the survey results on vaccination intentions indicate that 37.8% and 20.9% of participants reported being somewhat or very unlikely to receive the influenza and pneumococcal vaccines, respectively [7], and results from the 2021–2021 Seasonal Influenza Vaccination Coverage Survey suggest that the lack of awareness about pneumococcal vaccines is among the top three reasons for non-vaccination [9]. These findings of low coverage, intention, and awareness about recommended vaccinations warrant further research and public health efforts to improve vaccine uptake.

Increasing vaccine uptake to meet the vaccination coverage goals requires better understanding of populations and factors associated with lower vaccination coverage. Immigrants encounter unique personal, social, and physical challenges in becoming vaccinated and being an immigrant may be associated with lower uptake of influenza and pneumococcal vaccines [10–16]. However, there is limited evidence in the Canadian context regarding the disparities in coverage of both vaccinations among immigrants – commonly defined in literature by whether one is foreign-born or not [16–19]. Given that immigrants represent almost one in four individuals in Canada [20], this knowledge is critical to inform strategies to improve vaccine uptake and work toward achieving health equity.

Our study objectives were to: 1) estimate and compare coverage of both vaccinations by immigrant status, 2) quantify the association between immigrant status and vaccination, and 3) evaluate the relative importance of immigrant status in predicting the coverage of each vaccination in comparison to other determinants of vaccine uptake.

## Methods

### Study design

We conducted a cross-sectional secondary analysis of data from the Canadian Longitudinal Study on Aging (CLSA) Tracking and Comprehensive cohorts collected at baseline (2011–2015) and follow up 1 (FU1; 2015–2018). We analyzed data from FU1, which is the most recent data set available with vaccination information collected from all study participants, unless data were only available from the baseline survey (e.g., immigrant status).

### Data source and study population

The CLSA is a national longitudinal cohort study on adult development and aging composed of 2 cohorts – Tracking and Comprehensive – followed every 3 years. Individuals unable to respond in English or French were excluded [21]. Details about the CLSA study have been published [21–23] and are available on the CLSA website (clsa-elcv.ca).

Eligibility criteria for this study were: participation in FU1; aged 65 years or older at FU1; availability of data on self-reported influenza vaccine uptake in the previous 12 months, pneumococcal vaccine uptake ever, and immigrant status; residence in one of 10 provinces at FU1 (Tracking cohort) or in one of 7 provinces at which data collection sites are situated (Comprehensive cohort).

### Influenza and pneumococcal vaccination status

Outcomes were self-reported influenza and pneumococcal vaccination status. During FU1, participants were asked, “Have you had [a] flu shot in the last 12 months” and “… [a] pneumonia shot (pneumococcal vaccination) in your life”. We defined those who answered “yes” as vaccinated, those who answered “no” as unvaccinated, and those who answered “don’t know” or refused to answer as missing, consistent with previous literature [24, 25].

### Immigrant status

During the baseline survey, participants were asked “In what country were you born?” and “In what year did you first come to Canada to live?”. As defined by the CLSA and previous literature [17, 18], we categorized those who were born outside of Canada as immigrant, those who were born in Canada as non-immigrant, and those who did not answer as missing.

### Covariates

Based on previous literature and content expertise [24–33], the following groups of covariates obtained at FU1 were analyzed: sociodemographics, health status/access to healthcare, lifestyle or health behaviour, social support and activities, and environmental [34–36]. We also included sex at birth, race and ethnocultural background, highest level of education, religion, and language most spoken at home, which were measured at baseline (Table S1). Categories with largest sample sizes were chosen as the reference category for nominal variables with three or more categories.

### Statistical analyses

We described the coverage of both vaccinations and key characteristics of the study population by immigrant status using proportions and 95% confidence intervals (CI). Poisson regression models with robust standard errors were used to examine the associations of immigrant status and other key equity stratifiers with influenza and pneumococcal vaccination and estimate prevalence ratios (PR) and adjusted prevalence ratios (aPR) [37]. Survey weights developed by the CLSA were applied [38]. R packages survey and gtsummary were used [39, 40].

For objective 3 (importance of immigrant status compared to other variables in predicting the coverage of each vaccination), we used random forest classification, a non-parametric machine learning technique that predicts outcomes by constructing multiple decision trees, each trained on random subsets of data and variables [41]. This analysis complements traditional regression analyses; variable importance measures that quantify the predictive power of each variable included in the model can be generated without making the assumptions required for regression models. The final prediction was determined by aggregating the majority vote from the decision trees and importance was defined by the degree to which a variable contributed to predicting the outcome [41, 42]. Participants were split 80:20 into training and testing data sets, respectively, using stratified random sampling on variables sexual orientation and language most spoken at home. Using the training data, a random forest algorithm was developed by tenfold cross-validation. Random forest hyperparameters were tuned to maximize the discrimination performance of the model (Table S2). We analyzed the association between covariates and vaccination using two methods. First, we measured the mean decrease in accuracy (i.e. the accuracy with which the model decreases when values of a particular feature are permuted) [41, 43]. Second, we calculated Shapley additive explanations (SHAP) values [44, 45]. SHAP is a method used to explain the decisions of machine learning models by showing how much each variable contributes to a specific prediction [46]. The contributions of each variable to individual predictions, quantified by absolute SHAP values, were weighted using CLSA analytic survey weights and averaged to generate global variable importance measures [38, 46]. Model performance metrics were evaluated using the test dataset. Analyses were conducted in R (version 4.3.3) with caret, randomForest, and fastshap packages [44, 47, 48].

### Sample size and missing data

Objective 1 included all eligible participants (influenza vaccination *n* = 23,214; pneumococcal vaccination *n* = 22,235). For objective 2, we conducted complete case analyses as the total proportion of missingness was less than 10% among covariates included (i.e., age, sex at birth, race and ethnocultural background, highest level of education, total household income, province of residence, urbanicity of residence) (influenza vaccination *n* = 21,108; pneumococcal vaccination *n* = 20,230) [49]. For objective 3, as the proportion of missingness among all covariates analyzed exceeded 10%, random forest imputation technique was used to impute missing data among eligible participants. We used R package missForest for imputation [50].

### Sensitivity analyses

First, we analyzed coverage of influenza and pneumococcal vaccinations stratified by region of birth and years lived in Canada to examine heterogeneities within immigrants. Second, we repeated objective 2 using imputed data from objective 3, and conversely repeated objective 3 using complete data only. Lastly, we repeated objective 3 after removing correlated predictors which may influence variable importance measures. We assessed correlation between covariates using Cramer’s V. After identifying three variable pairs with Cramer’s V of 0.5 or higher, we removed one variable from each pair guided by previous literature and clinical expertise.

## Results

### Study population

A total of 23,214 participants were eligible for influenza vaccination analyses (18,776 non-immigrants and 4,438 immigrants) and 22,235 participants for pneumococcal vaccination analyses (18,013 non-immigrants and 4,222 immigrants). Differences in immigrant and non-immigrant participants’ sociodemographic characteristics included the distribution of race and ethnocultural background, highest level of education, total household income, language most spoken at home, province, and urbanicity (Tables S3 and S4).

### Influenza and pneumococcal vaccination coverage

Coverage of influenza vaccination in the past 12 months was lower among immigrant participants (63.8%, 95% CI: 60.9–66.7%) compared to non-immigrant participants (66.9%, 95% CI: 65.5–68.3%) (Table 1). Overall, the proportion of those who had ever received a pneumococcal vaccine was lower than the proportion of those who received an influenza vaccine within the previous 12 months, regardless of immigrant status (Table 2). Coverage of a pneumococcal vaccination was also lower among immigrant participants (48.7%, 95% CI: 45.6–51.8%) compared to non-immigrant participants (55.8%, 95% CI: 54.3–57.3%), and the difference in coverage between the two groups was greater for pneumococcal vaccination than for influenza vaccination.

**Table 1:**
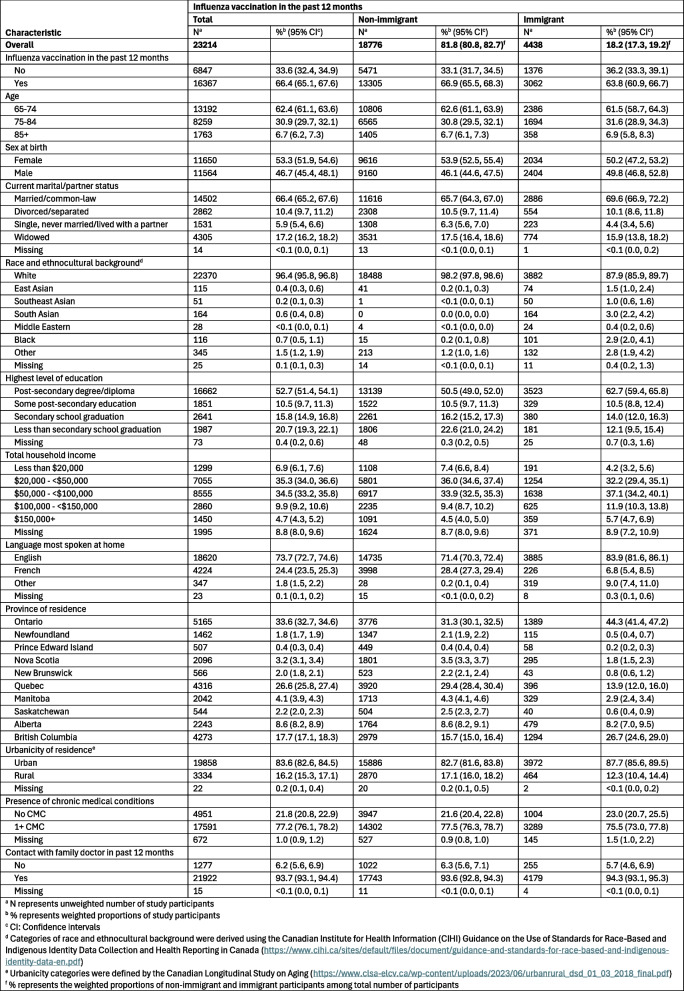
Key characteristics and self-reported influenza vaccination in the past 12 months of the Canadian Longitudinal Study on Aging (CLSA) participants at follow up 1 (2015-2018) by immigrant status

**Table 2:**
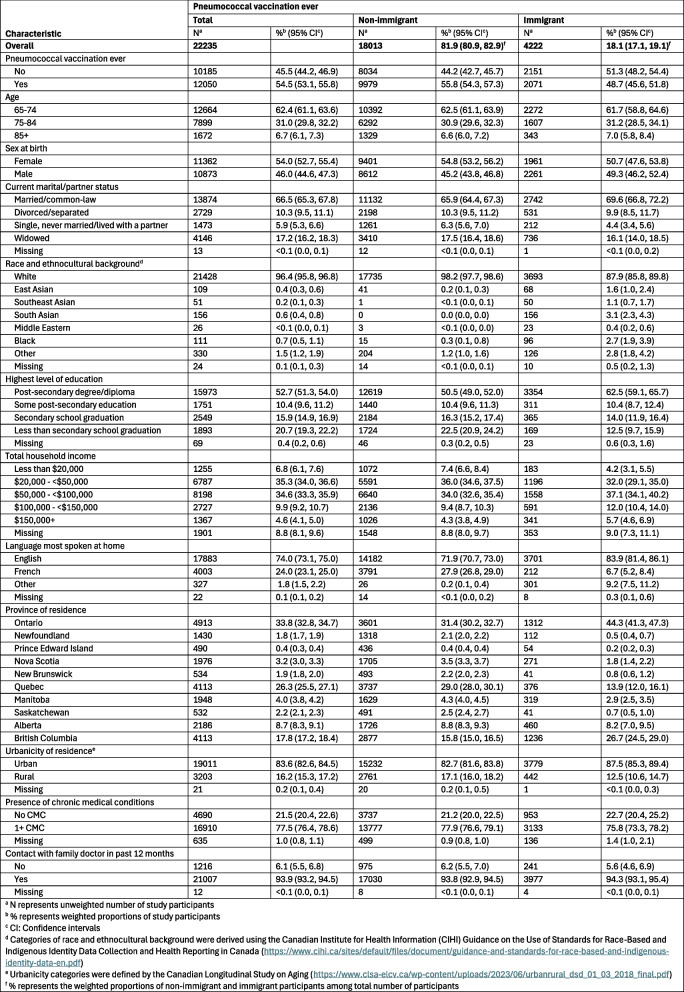
Key characteristics and self-reported pneumococcal vaccination ever of CLSA participants at follow up 1 (2015-2018) by immigrant status

### Association between immigrant status and vaccination coverage

Table 3 presents the results of the robust Poisson regression models. Prevalence of influenza vaccination was lower among immigrant compared to non-immigrant participants (aPR: 0.92, 95% CI: 0.89–0.95). Age, total household income, province, and urbanicity were associated with influenza vaccination. Residents of Quebec reported lowest vaccination rates, followed by Newfoundland, Saskatchewan, and British Columbia, while residents of Nova Scotia reported the highest influenza vaccination coverage.

**Table 3:**
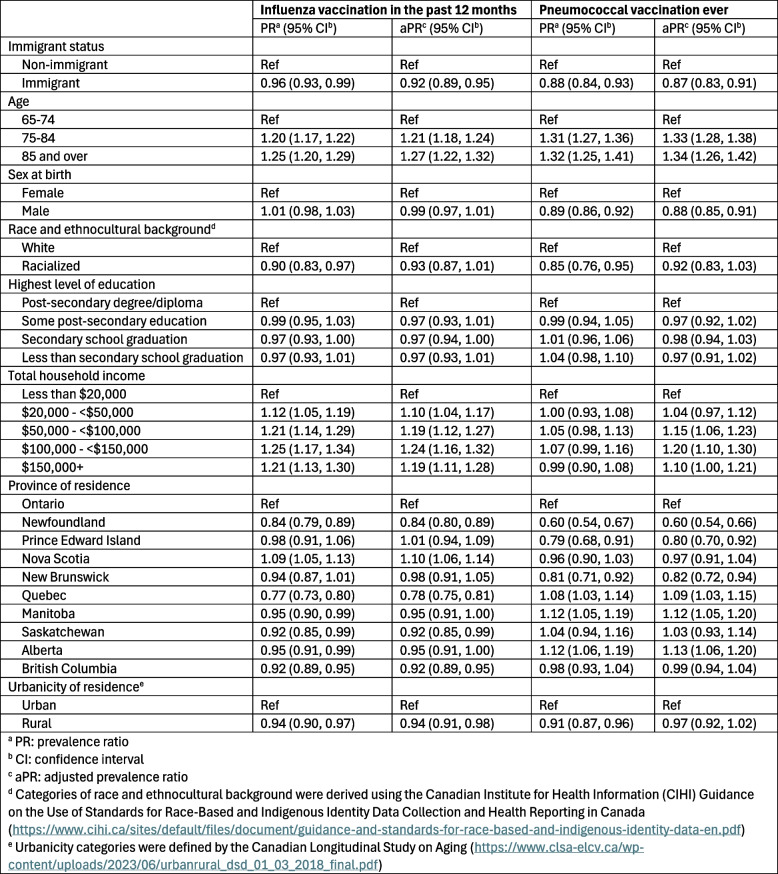
Prevalence of influenza and pneumococcal vaccination in CLSA participants at follow up 1 (2015-2018)

Prevalence of pneumococcal vaccination was also lower among immigrant compared to non-immigrant participants (aPR: 0.87, 95% CI: 0.83–0.91). Age, sex at birth, total household income, and province were associated with vaccination. While provincial variations in uptake remained, residents of Newfoundland reported lowest coverage, followed by Prince Edward Island and New Brunswick, whereas residents of Alberta, Manitoba, and Quebec reported the highest pneumococcal vaccination coverage.

### Ranking of determinants of vaccination coverage using random forest algorithms

**Fig. 1:**
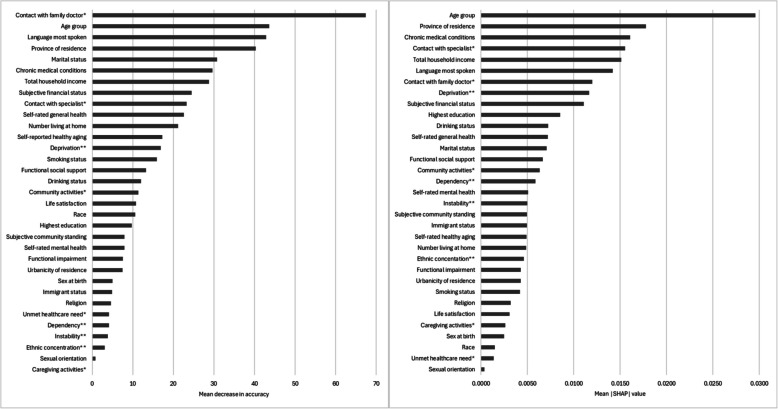
Ranking of determinants of CLSA participants’ self-reported influenza vaccination in the past 12 months at follow up 1 by mean decrease in accuracy (MDA; left) and mean absolute SHapley Additive exPlanations (SHAP; right) values. MDA values represent the mean extent (in percentage points) to which the model’s accuracy decreases when values of a feature are permuted, and range between 0 and 100. SHAP values represent the weighted mean absolute change in predicted probability of outcome, expressed as the average magnitude of the contribution that a variable makes to individual predictions, and range between 0 and 1. * In the past 12 months. ** Dimensions of the Canadian Marginalization Index (i.e., quintile of dependency/deprivation/ethnic concentration/instability)

Figure 1 shows the ranking of determinants of influenza vaccination. Based on variables’ values of MDA, contact with a family doctor in the past 12 month was the most important predictor of influenza vaccination coverage (MDA: 67.4), suggesting that if values of this variable were permuted, then the model’s accuracy would decrease by 67.4 percentage points (pp) on average. Variables’ mean absolute SHAP values indicate that age group was the most important predictor of influenza vaccination coverage (SHAP: 0.030), suggesting that on average, age group contributed to 0.030 (3.0 pp) change in predicted probability of influenza vaccination for each participant. While there were differences in results based on the importance measure used (i.e., MDA or SHAP), the results indicated that the most important predictors of influenza vaccination coverage included age (MDA: 43.6; SHAP: 0.030), province (MDA: 40.3; SHAP: 0.018), language most spoken at home (MDA: 42.8; SHAP: 0.014), presence of chronic medical conditions (MDA: 29.7; SHAP: 0.016), and contact with a family doctor (MDA: 67.4; SHAP: 0.012) and medical specialist (MDA: 23.3; SHAP: 0.016) in the previous 12 months.

**Fig. 2:**
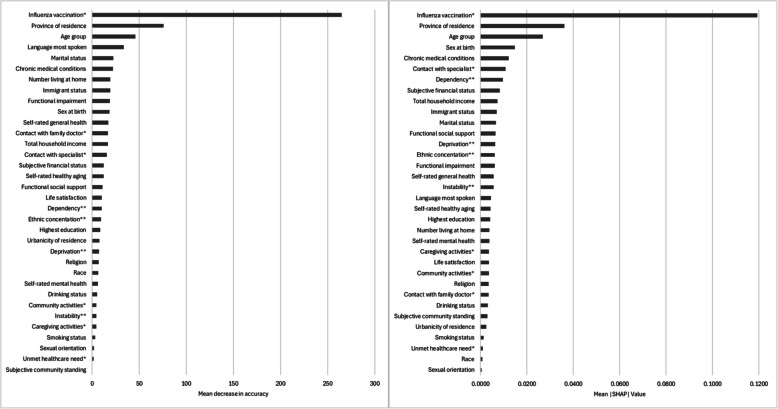
Ranking of determinants of CLSA participants’ self-reported pneumococcal vaccination ever at follow up 1 by mean decrease in accuracy (MDA; left) and mean absolute SHapley Additive exPlanations (SHAP; right) values. MDA values represent the mean extent (in percentage points) to which the model’s accuracy decreases when values of a feature are permuted, and range between 0 and 100. SHAP values represent the weighted mean absolute change in predicted probability of outcome, expressed as the average magnitude of the contribution that a variable makes to individual predictions, and range between 0 and 1. * In the past 12 months. ** Dimensions of the Canadian Marginalization Index (i.e., quintile of dependency/deprivation/ethnic concentration/instability)

Figure 2 shows the ranking of determinants of pneumococcal vaccination. Both measures of importance (i.e., MDA and SHAP) indicated that the most important predictor of pneumococcal vaccination was influenza vaccination in the previous 12 months (MDA: 265; SHAP: 0.119), suggesting that on average, if values of this variable were permuted, then the models’ accuracy would decrease by 265 pp, and that the variable contributed to 0.119 (11.9 pp) change in predicted probability of pneumococcal vaccination for each participant. The second and third most important predictors were province (MDA: 75.7; SHAP: 0.036) and age (MDA: 45.8; SHAP: 0.027), respectively. In contrast to its relatively low ranking among determinants of influenza vaccination coverage (MDA: 4.85; SHAP: 0.005), immigrant status was among the 10 most important predictors of pneumococcal vaccination coverage (MDA: 19.2; SHAP: 0.007). Random forest model performance metrics are presented in Table S5.

### Sensitivity analyses

Analyses using participants’ region of birth revealed heterogeneities in influenza and pneumococcal vaccination coverage among immigrants. In comparison to non-immigrants, participants born in other regions reported lower influenza and pneumococcal vaccination coverage, except those born in other North American countries for influenza vaccination (Figure S1). Influenza and pneumococcal vaccination coverage were generally positively associated with number of years lived in Canada among immigrants; immigrants who lived in Canada for 61 years or more reported comparable coverage of both vaccines to non-immigrants (Figure S2). Results of other sensitivity analyses were similar to those of the main analyses (Figures S3-S6 and Table S6).

## Discussion

In our cross-sectional analysis of 2015–2018 CLSA data, we found that self-reported influenza and pneumococcal vaccination coverage were lower among immigrants compared to non-immigrants, and that the difference was greater for pneumococcal vaccination. Immigrant status was among the least important predictors of influenza vaccination, but among the most important predictors of pneumococcal vaccination.

Our findings of lower vaccination coverage in immigrant compared to non-immigrant older adults are consistent with the limited literature on influenza and pneumococcal vaccination coverage in Canada [13, 14] and in the US [51], although the association between immigrant status and influenza and pneumococcal vaccination coverage remains inconclusive [27, 52]. Potential reasons for lower vaccination coverage among immigrants include personal thoughts, beliefs, and concerns about vaccines, as well as those rooted in historical and structural barriers that challenge vulnerable groups like older adult immigrants from accessing vaccinations. These include lack of vaccine information and awareness, mistrust in vaccines and the wider governance and healthcare system, language barriers, and racism [15, 16, 53, 54]. Cost and affordability of vaccinations are also commonly identified as barriers to vaccinations among immigrants [15, 16, 53], although this may be less applicable in our study as influenza and pneumococcal vaccinations are publicly funded for adults aged 65 years and older in all jurisdictions in Canada [6, 55]. The discrepancies in public funding of vaccinations may contribute to inconclusive associations between immigrant status and vaccination coverage reported in literature [27, 52].

We found that the association between immigrant status and vaccination differed between influenza and pneumococcal vaccines. Disparities were larger for pneumococcal vaccination and immigrant status was a more important predictor for pneumococcal vaccination than for influenza vaccination. One reason for these differences may be availability and accessibility of vaccinations (i.e., how readily available and accessible vaccinations are to those wishing to become vaccinated). In Canada, depending on the province and/or territory of residence, influenza vaccines are available for free in various settings (i.e., doctors’ offices, pharmacies, and public health sites), whereas pneumococcal vaccines are not as widely available [56]. Research suggests that over 50% of adults who received an influenza vaccine during the 2023–2024 influenza season received their vaccinations at pharmacies [57], and that administration of influenza vaccines by pharmacists increased vaccination coverage by improving availability, proximity, and accommodation of vaccinations [58]. This suggests that expanding availability of vaccines with low coverage, such as pneumococcal vaccinations, for administration in various settings, including pharmacies, may improve vaccination coverage and disparities. Furthermore, these differences in accessibility/availability of vaccinations at different settings, as well as differences in vaccination programs (particularly for pneumococcal) may contribute to the large provincial differences observed in influenza and pneumococcal vaccination coverage. For example, unlike influenza vaccinations which are consistently publicly funded for all adults aged 65 years and older in all provinces and territories, pneumococcal vaccination schedules differ by the type of vaccine offered and eligibility (e.g., whether one has previously received a pneumococcal vaccine or not) across jurisdictions. Another reason for the differences may be awareness of vaccines. A 2021 survey among Canadian adults aged 65 years and older reported that the “doctor did not mention it” or that they “never heard of this vaccine” as the leading reasons for pneumococcal non-vaccination [9]. The importance of influenza vaccines are promoted through annual vaccination campaigns and numerous advocacy efforts [59]. As such, disparities in coverage by immigrant status may be smaller for influenza vaccination compared to pneumococcal vaccination due to heightened efforts to improve accessibility and awareness of influenza vaccinations, which are barriers to vaccinations experienced by immigrant communities. Thus, public health efforts to increase awareness of and accessibility to vaccines may be effective in increasing vaccine uptake in the population, but a “one size fits all” approach may not be appropriate for the complex and diverse needs of diverse older adults [15].

The importance of a variable in predicting an outcome reflects its predictive ability, which is influenced by how the variable is defined. The low relative ranking of immigrant status in predicting influenza vaccination coverage may suggest that the study’s definition of this variable cannot sufficiently capture important heterogeneities within immigrant communities by factors such as recency of immigration, immigration category, region of birth, and generation, that influence immigrants’ influenza vaccination decisions [19, 60, 61]. In fact, our sensitivity analyses revealed that coverage of both vaccinations differed considerably among immigrants by region of birth and time since immigration.

Overall, our findings reveal disparities in influenza and pneumococcal vaccination coverage among older immigrant adults living in Canada, likely driven by both individual and structural barriers. Further research on vaccination coverage using more disaggregated definitions and categorizations of immigrants is warranted. Furthermore, immigrant health is determined simultaneously by the interaction of various social positions and dimensions of inequalities [62, 63]. Therefore, future studies should consider how intersecting social factors, historical inequities, and vaccine-specific issues influence decision-making, particularly among immigrants, to better address these compounded barriers and improve vaccine uptake equitably in Canada [64, 65].

### Limitations

The characteristics of the CLSA cohort may limit generalizability of results, because of overrepresentation of individuals born in Canada and with higher socioeconomic status, education, and better health compared to the general older adult population in Canada [21, 22]. Further, vaccination coverage reported by previous studies on influenza and pneumococcal vaccination using CLSA data indicate that coverage of both vaccinations among study participants are higher than those of older adults in Canada overall [24, 25, 66]. Our definition of immigrant status may have masked important heterogeneities within immigrant communities. Sensitivity analyses by region of birth and years lived in Canada were conducted, but analyses were limited due to small sample sizes. We analyzed data from the first follow-up, which was collected before the COVID-19 pandemic. It is unknown whether the association between immigrant status and vaccination coverage has changed during the pandemic, during which there were extensive campaigns to promote vaccination awareness and accessibility [67, 68]. Vaccination data were self-reported, which may not be accurate. However, previous analyses using self-reported influenza and pneumococcal vaccination data suggest that this would have minimal impact [24, 69, 70]. Lastly, we lacked data on reasons for vaccination or non-vaccination, which limited our ability to explore decision-making factors that differ between immigrants and non-immigrants.

## Conclusions

We found lower self-reported coverage of an influenza vaccination in the previous 12 months and pneumococcal vaccination ever among immigrants compared to non-immigrants. The disparities in coverage were greater for pneumococcal vaccination than for influenza vaccination. Immigrant status was also found to be an important predictor of pneumococcal vaccination, while not of influenza vaccination. Future studies should investigate the coverage of and reasons for uptake of various vaccines in immigrants using more disaggregated definitions and categorizations and accounting for intersecting vulnerabilities to gain a deeper understanding of the heterogeneous vaccination experiences within immigrant communities.

## Supplementary Information

Supplementary Material 1.

## Data Availability

Data analyzed in the current study are available from the Canadian Longitudinal Study on Aging for approved researchers who meet the criteria for access. The datasets are not publicly available and users must sign an agreement that prohibits sharing of the data beyond the approved research team. Further information about data access and application can be found at [www.clsa-elcv.ca] (http:/www.clsa-elcv.ca).

## Abbreviations

aPR: Adjusted prevalence ratio
CI: Confidence intervals
CLSA: The Canadian Longitudinal Study on Aging
FU1: Follow-up 1
NACI: National Advisory Committee on Immunization
pp: Percentage points
PR: Prevalence ratio
SHAP: Shapley additive explanations
