# Supplementary material for: Coverage of the influenza and pneumococcal vaccinations among immigrant and non-immigrant older adults in Canada: a cross-sectional analysis of data from the Canadian Longitudinal Study on Aging (CLSA)
Source: BMC Public Health. 2025 Nov 4;25:3775. doi: 10.1186/s12889-025-25005-z (PMC12584234; doi:10.1186/s12889-025-25005-z)
Supplement: Supplementary file 1 — Supplementary Material 1. [file 12889_2025_25005_MOESM1_ESM.pdf]

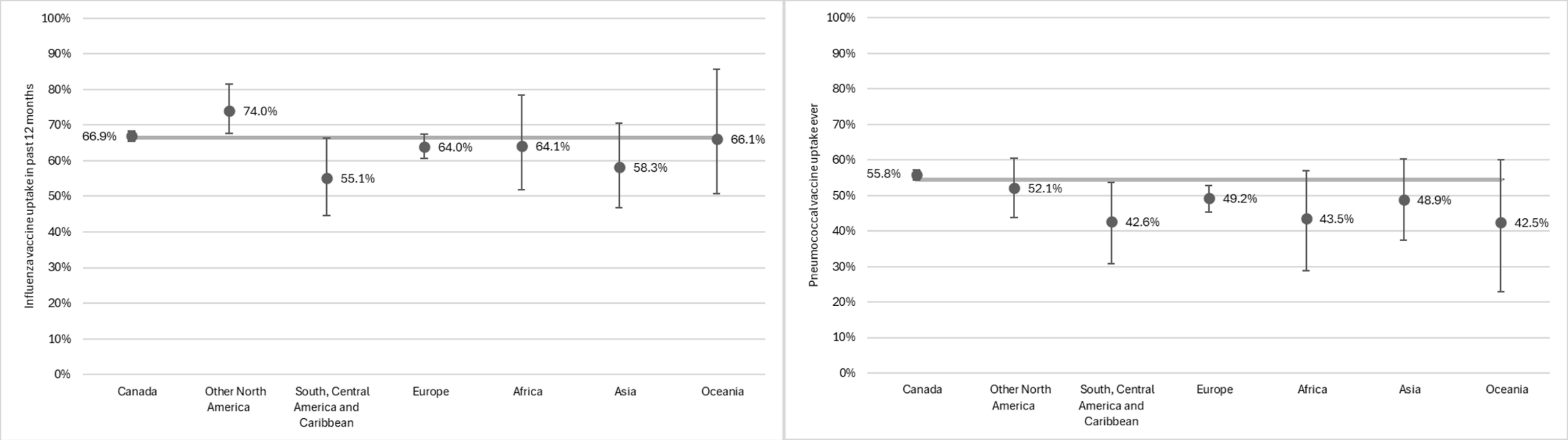

Figure S1. Self-reported influenza vaccination in the past 12 months (left) and pneumococcal vaccination ever (right) of CLSA participants at follow up 1 (2015-2018) by region of birth. Grey line represents self-reported vaccination coverage among all eligible study participants (influenza vaccination in the past 12 months: 66.4% [95% CI: 65.1-67.6]; pneumococcal vaccination ever: 54.5% [95% CI: 53.1-55.8]).

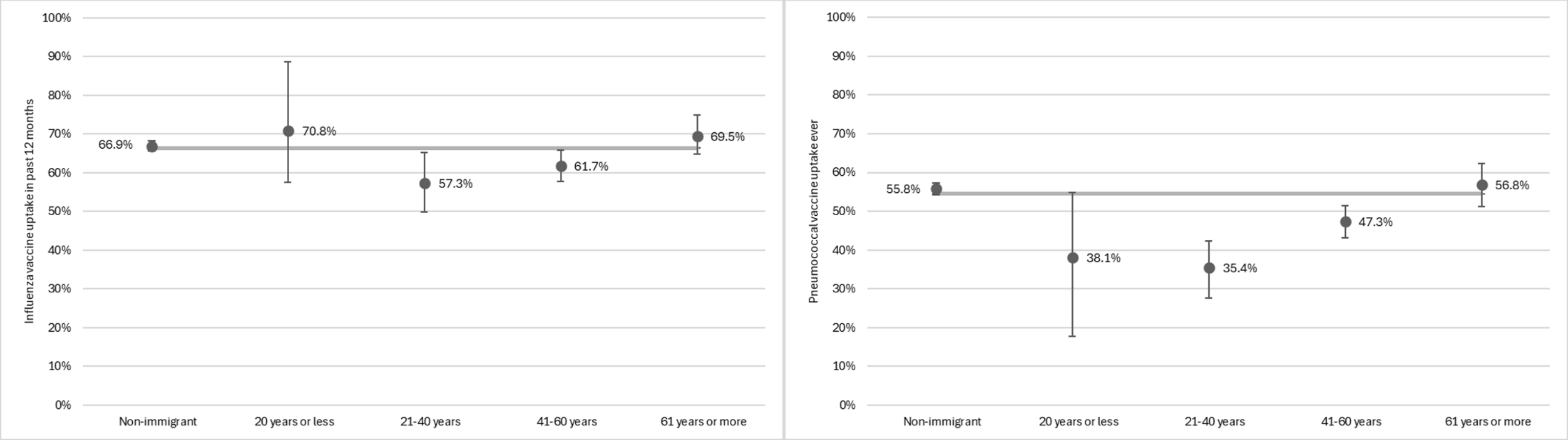

Figure S2. Self-reported influenza vaccination in the past 12 months (left) and pneumococcal vaccination ever (right) of CLSA participants at follow up 1 (2015-2018) by years lived in Canada. Grey line represents self-reported vaccination coverage among all eligible study participants (influenza vaccination in the past 12 months: 66.4% [95% CI: 65.1-67.6]; pneumococcal vaccination ever: 54.5% [95% CI: 53.1-55.8]).

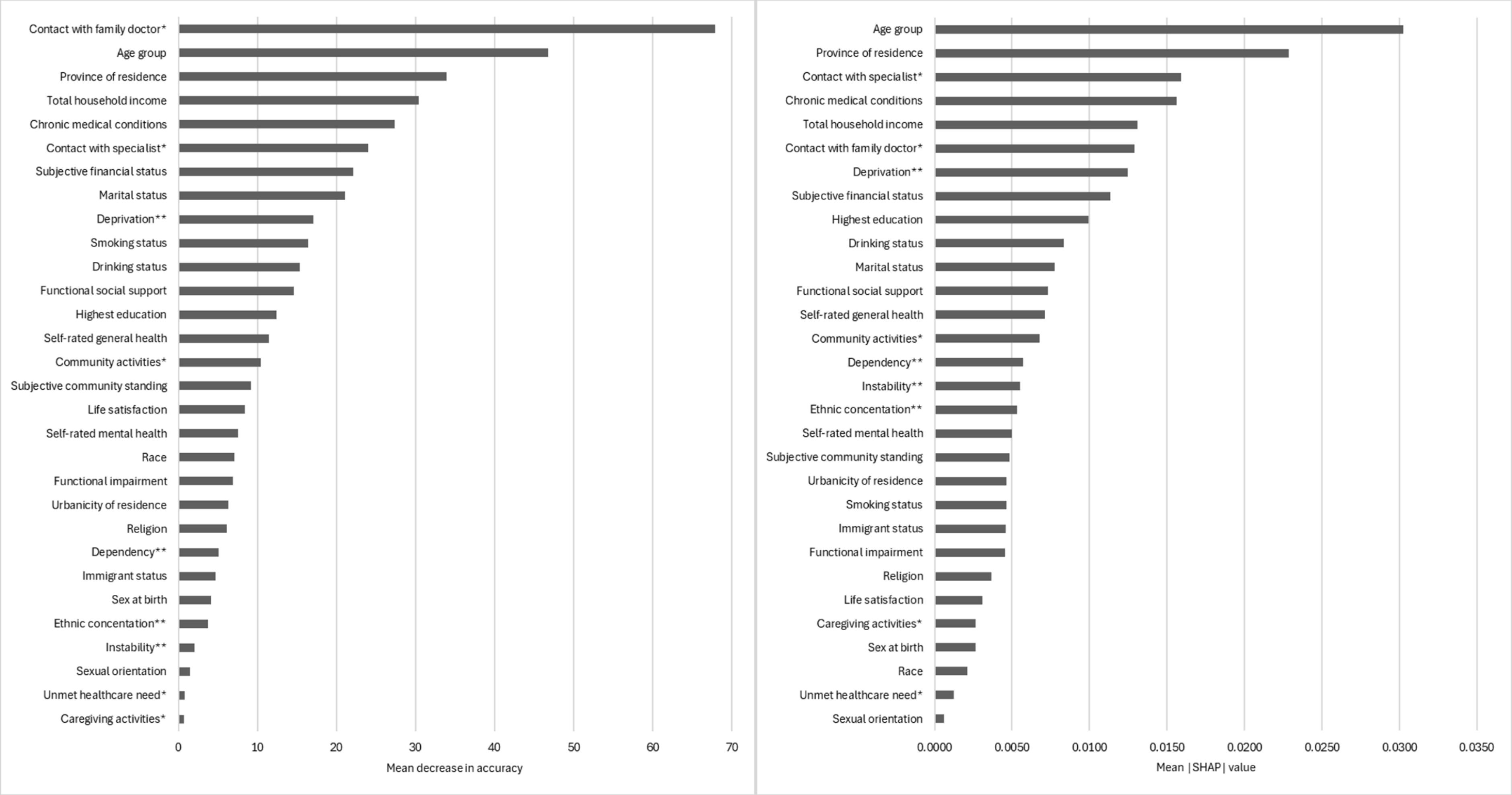

Figure S3. Ranking of determinants of CLSA participants' self-reported influenza vaccination in the past 12 months at follow up 1 by mean decrease in accuracy (MDA; left) and mean absolute Shapley additive explanations (SHAP; right) values using imputed data without correlated variables. MDA values represent the mean extent (in percentage points) to which the model's accuracy decreases when values of a feature are permuted, and range between 0 and 100. SHAP values represent the weighted mean absolute change in predicted probability of outcome, expressed as the average magnitude of the contribution that a variable makes to individual predictions, and range between 0 and 1.

* In the past 12 months

** Dimensions of the Canadian Marginalization Index (i.e., quintile of dependency/deprivation/ethnic concentration/instability)

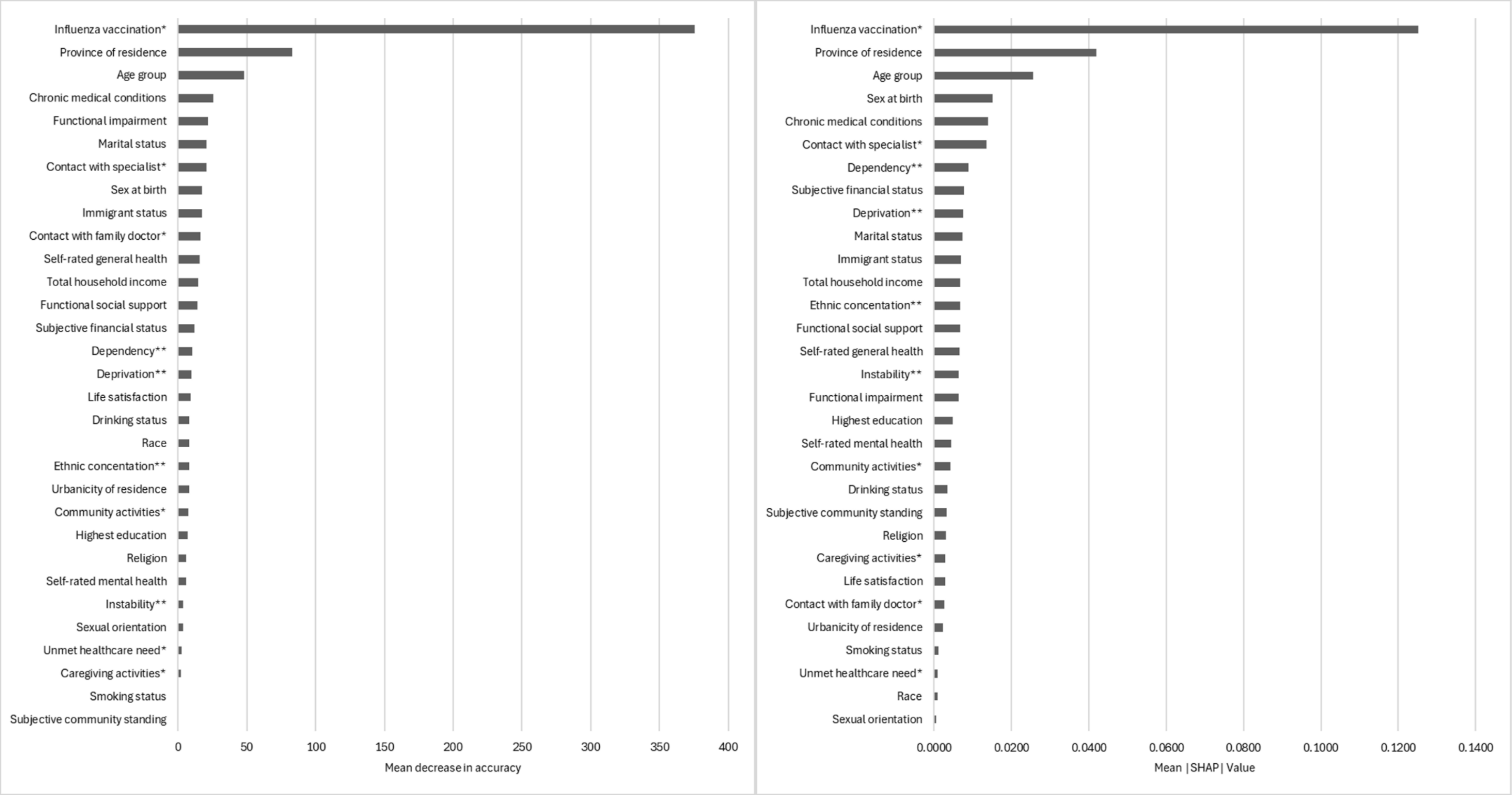

Figure S4. Ranking of determinants of CLSA participants' self-reported pneumococcal vaccination ever at follow up 1 by mean decrease in accuracy (MDA; left) and mean absolute Shapley additive explanations (SHAP; right) values using imputed data without correlated variables. MDA values represent the mean extent (in percentage points) to which the model's accuracy decreases when values of a feature are permuted, and range between 0 and 100. SHAP values represent the weighted mean absolute change in predicted probability of outcome, expressed as the average magnitude of the contribution that a variable makes to individual predictions, and range between 0 and 1.

* In the past 12 months

** Dimensions of the Canadian Marginalization Index (i.e., quintile of dependency/deprivation/ethnic concentration/instability)

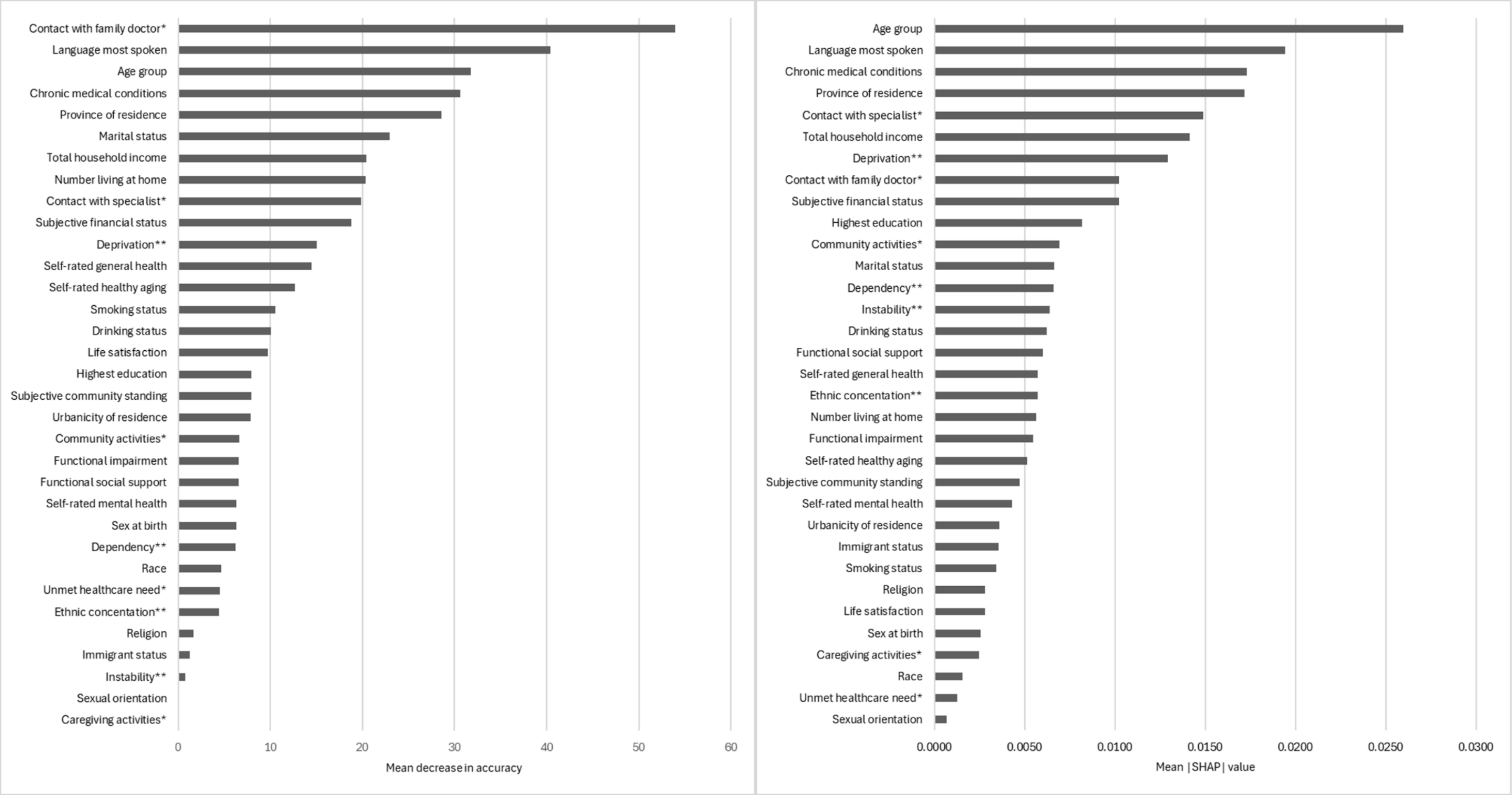

Figure S5. Ranking of determinants of CLSA participants' self-reported influenza vaccination in the past 12 months at follow up 1 by mean decrease in accuracy (MDA; left) and mean absolute Shapley additive explanations (SHAP; right) values using complete data with no missing observations. MDA values represent the mean extent (in percentage points) to which the model's accuracy decreases when values of a feature are permuted, and range between 0 and 100. SHAP values represent the weighted mean absolute change in predicted probability of outcome, expressed as the average magnitude of the contribution that a variable makes to individual predictions, and range between 0 and 1.

* In the past 12 months

** Dimensions of the Canadian Marginalization Index (i.e., quintile of dependency/deprivation/ethnic concentration/instability)

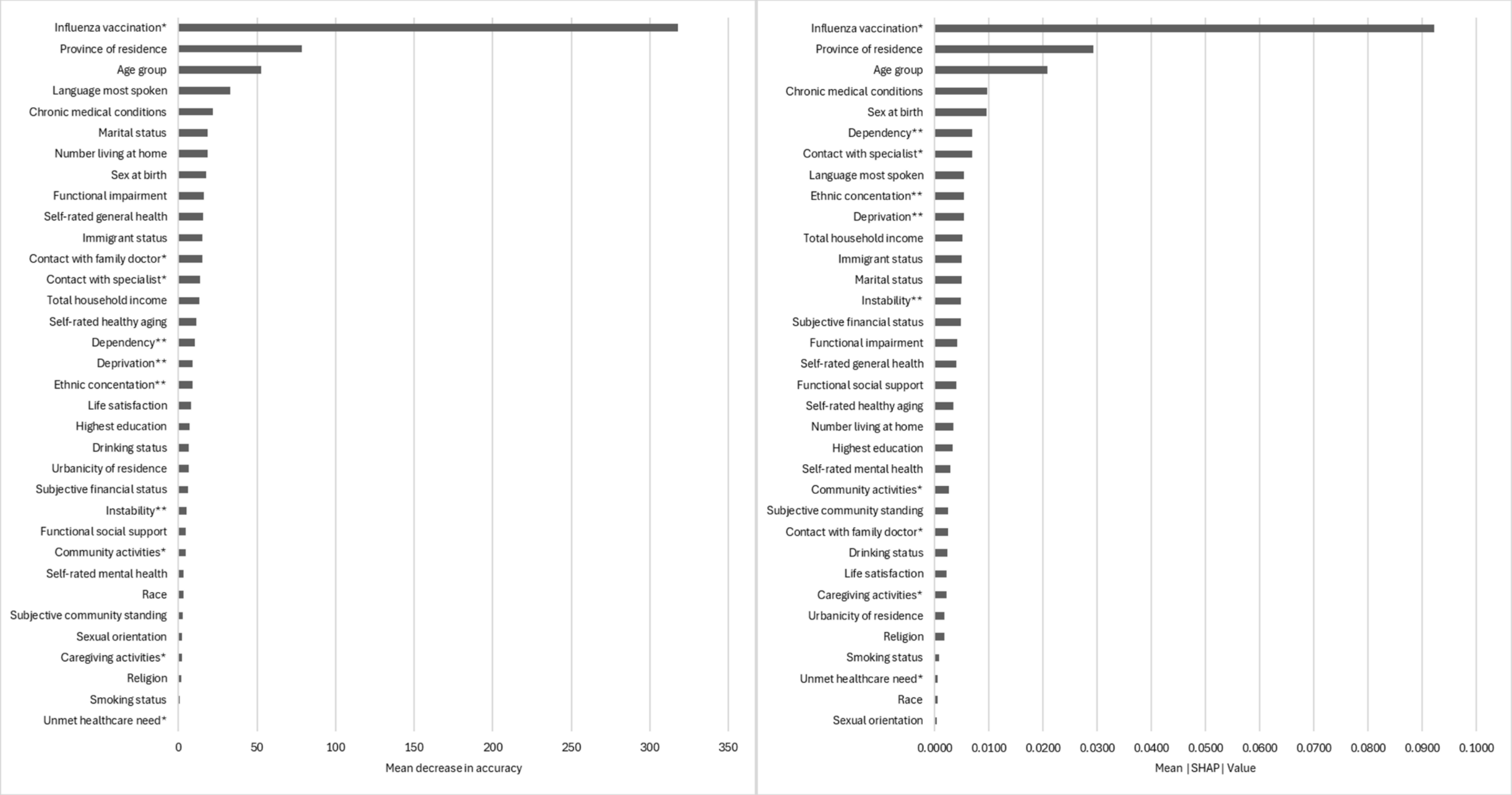

Figure S6. Ranking of determinants of CLSA participants' self-reported pneumococcal vaccination ever at follow up 1 by mean decrease in accuracy (MDA; left) and mean absolute Shapley additive explanations (SHAP; right) values using complete data with no missing observations. MDA values represent the mean extent (in percentage points) to which the model's accuracy decreases when values of a feature are permuted, and range between 0 and 100. SHAP values represent the weighted mean absolute change in predicted probability of outcome, expressed as the average magnitude of the contribution that a variable makes to individual predictions, and range between 0 and 1.

* In the past 12 months

** Dimensions of the Canadian Marginalization Index (i.e., quintile of dependency/deprivation/ethnic concentration/instability)

Table S1. CLSA variable names and categorization of variables for analyses.

Variable	CLSA variable name	Categorization for analysis	Categorization for objective 3 (if different)
**Outcome**			
Self-reported influenza vaccination	PHB_FLUV_COF1<br>PHB_FLUV_TRF1	Unvaccinated<br>Vaccinated<br>Missing	
Self-reported pneumococcal vaccination	PHB_PCV_COF1<br>PHB_PCV_TRF1	Unvaccinated<br>Vaccinated<br>Missing	
**Primary predictor**			
Immigrant status	SDC_FIMM_COM<br>SDC_FIMM_TRM	Non-immigrant<br>Immigrant<br>Missing	
**Socio-demographic and -economic**			
Age group	AGE_NMBR_COF1<br>AGE_NMBR_TRF1	65-74 years old<br>75-84 years old<br>85 years and older<br>Missing	
Sex at birth	SEX_ASK_COM<br>SEX_ASK_TRM	Female<br>Male	
Sexual orientation	SDC_ORTN_COF1<br>SDC_ORTN_TRF1	Heterosexual<br>Homosexual<br>Bisexual<br>Other<br>Missing	Heterosexual<br>Bisexual, homosexual, or other<br>Missing
Current marital/partner status	SDC_MRTL_COF1<br>SDC_MRTL_TRF1	Married/common-law relationship<br>Divorced/separated<br>Single, never married/lived with a partner<br>Widowed<br>Missing	
Race and ethnocultural background	SDC_DCGT_COM<br>SDC_DCGT_TRM	White<br>East Asian<br>Southeast Asian<br>Middle Eastern<br>Black<br>Other<br>Missing	White<br>Racialized<br>Missing<br><br>**This variable was re-categorized for objectives 2 and 3*

Highest level of education	ED_UDR04_COM<br>ED_UDR04_TRM	Post-secondary degree/diploma<br>Some post-secondary education<br>Secondary school graduation<br>Less than secondary school graduation<br>Missing	
Total household income	INC_TOT_COF1<br>INC_TOT_TRF1	Less than $20,000<br>$20,000 - <$50,000<br>$50,000 - <$100,000<br>$100,000 - <$150,000<br>$150,000+<br>Missing	
Religion	SDC_RELG_COM<br>SDC_RELG_TRM	Christianity<br>Other<br>None<br>Missing	
Language most spoken at home	SDC_LGMST_COM<br>SDC_LGMST_TRM	English<br>French<br>Other<br>Missing	
**Perceived socio-economic standing**			
Subjective financial situation	WEA_FNSTATUS_COF1<br>WEA_FNSTATUS_TRF1	Manage very well<br>Manage quite well<br>Get by alright<br>Don't manage very well/have difficulties<br>Missing	Manage very well<br>Manage quite well<br>Get by alright/don't manage well/have difficulties<br>Missing
Subjective standing in community	SEQ_LADSCALE_COF1<br>SEQ_LADDER_TRF1	Very high<br>High<br>Medium<br>Low<br>Very low<br>Missing	High<br>Medium<br>Low<br>Missing
**Health status/access to healthcare**			

Presence of chronic medical conditions (influenza)	Cardiovascular: 1) heart disease (CCC_HEART_COF1, CCT_HEART_TRF1); 2) heart attack/MI (CCC_AMI_COF1, CCT_AMI_TRF1); 3) high BP/hypertension (CCC_HBP_COF1, CCT_HBP_TRF1)<br>Respiratory: 1) chronic lung disease (CCC_COPD_COF1, CCT_COPD_TRF1); 2) asthma (CCC_ASTHM_COF1, CCT_ASTHM_TRF1)<br>Cerebrovascular: 1) stroke (CCC_CVA_COF1, CCT_CVA_TRF1); 2) mini-stroke/TIA (CCC_TIA_COF1, CCT_TIA_TRF1)<br>Renal: 1) kidney disease/failure (CCC_KIDN_COF1, CCT_KIDN_TRF1)<br>Diabetes: 1) diabetes (DIA_DIAB_COF1, CCT_DIAB_TRF1)<br>Cancer: 1) cancer (CCC_CANC_COF1, CCT_CANC_TRF1)<br>Neurologic: 1) Alzheimer's (CCC_ALZH_COF1, CCT_ALZH_TRF1); 2) Parkinson's (CCC_PARK_COF1, PKD_PARK_TRF1)	No chronic medical conditions<br>1 or more chronic medical conditions<br>Missing	

Presence of chronic medical conditions (pneumonia)	Cardiovascular: 1) heart disease (CCC_HEART_COF1, CCT_HEART_TRF1); 2) heart attack/MI (CCC_AMI_COF1, CCT_AMI_TRF1); 3) angina (CCC_ANGI_COF1, CCT_ANGI_TRF1); 4) high BP/hypertension (CCC_HBP_COF1, CCT_HBP_TRF1)<br>Respiratory: 1) chronic lung disease (CCC_COPD_COF1, CCT_COPD_TRF1); 2) asthma (CCC_ASTHM_COF1, CCT_ASTHM_TRF1)<br>Cerebrovascular: 1) stroke (CCC_CVA_COF1, CCT_CVA_TRF1); 2) mini-stroke/TIA (CCC_TIA_COF1, CCT_TIA_TRF1)<br>Renal: 1) kidney disease/failure (CCC_KIDN_COF1, CCT_KIDN_TRF1)<br>Diabetes: 1) diabetes (DIA_DIAB_COF1, CCT_DIAB_TRF1)<br>Cancer: 1) cancer (CCC_CANC_COF1, CCT_CANC_TRF1)<br>Neurologic: 1) Alzheimer's (CCC_ALZH_COF1, CCT_ALZH_TRF1); 2) Parkinson's (CCC_PARK_COF1, PKD_PARK_TRF1); 3) multiple sclerosis (CCC_MS_COF1, CCT_MS_TRF1)	No chronic medical conditions<br>1 or more chronic medical conditions<br>Missing	
Functional impairment	ADL_DCLS_COF1<br>ADL_DCLS_TRF1	No functional impairment<br>Some impairment<br>Missing	
Unmet healthcare need in the past 12 months	MET_NEED_COF1<br>MET_NEED_TRF1	No<br>Yes<br>Missing	
Contact with a family doctor in the past 12 months	HCU_FAMPHY_COF1<br>HCU_FAMPHY_TRF1	No<br>Yes<br>Missing	
Contact with a medical specialist in the past 12 months	HCU_SPEC_COF1<br>HCU_SPEC_TRF1	No<br>Yes<br>Missing	
**Perceived health status**			

Self-rated general health	GEN_HLTH_COF1<br>GEN_HLTH_TRF1	Excellent<br>Very good<br>Good<br>Fair<br>Poor<br>Missing	Excellent<br>Very good<br>Good<br>Fair/poor<br>Missing
Self-rated mental health	GEN_MNTL_COF1<br>GEN_MNTL_TRF1	Excellent<br>Very good<br>Good<br>Fair<br>Poor<br>Missing	Excellent<br>Very good<br>Good<br>Fair/poor<br>Missing
Self-rated healthy aging	GEN_OWNAG_COF1<br>GEN_OWNAG_TRF1	Excellent<br>Very good<br>Good<br>Fair<br>Poor<br>Missing	Excellent<br>Very good<br>Good<br>Fair/poor<br>Missing
**Lifestyle or health behavior**			
Current smoking status	SMK_CURRCG_COF1<br>SMK_CURRCG_TRF1	Not at all<br>Occasionally<br>Daily<br>Missing	Not at all<br>Yes (occasionally/daily)<br>Missing
Current drinking status	ALC_TTM_COF1<br>ALC_TTM_TRF1	Did not drink in the last 12 months<br>Occasional drinker<br>Regular drinker<br>Missing	
Flu vaccination in the past 12 months (PCV only)	PHB_FLUV_COF1<br>PHB_FLUV_TRF1	Unvaccinated<br>Vaccinated<br>Missing	
Satisfaction with life	SLS_DCLS_COF1<br>SLS_DCLS_TRF1	Satisfied/extremely satisfied<br>Neutral or slightly dis/satisfied<br>Extremely dissatisfied/dissatisfied<br>Missing	
**Social support and activities**			
Number of people living in household excluding participant	SN_LIVH_NB_COF1<br>SN_LIVH_NB_TRF1	No other person living in household<br>1 other person living in household<br>2+ other persons living in household<br>Missing	

Functional social support	SSA_DPALL_COF1<br>SSA_DPALL_TRF1	High social support<br>Medium-high social support<br>Medium-low social support<br>Low social support<br>Missing	
Participation in providing assistance/caregiving in the past 12 months	CAG_FPAS_COF1<br>CAG_FPAS_TRF1	Did not provide any assistance<br>Did provide assistance<br>Missing	
Participation in community-related activities in the past 12 months	SPA_DFRE_COF1<br>SPA_DFRE_TRF1	Did not participate/participated once a year<br>Participated at least once a month<br>Participated at least once a week<br>Participated at least once a day<br>Missing	Did not participate/participated once a year/month<br>Participated at least once a week<br>Participated at least once a day<br>Missing
**Environmental**			
Province of residence	WGHTS_PROV_COF1<br>WGHTS_PROV_TRF1	Ontario<br>Newfoundland<br>Prince Edward Island<br>Nova Scotia<br>New Brunswick<br>Quebec<br>Manitoba<br>Saskatchewan<br>Alberta<br>British Columbia<br>Missing	Ontario<br>Maritimes<br>Quebec<br>Prairies<br>British Columbia
Urbanicity of residence	SDC_URBAN_RURAL_COF1<br>SDC_URBAN_RURAL_TRF1	Urban<br>Rural<br>Missing	
Quintile of instability	CMG16_06_COF1<br>CMG16_06_TRF1	Least unstable<br>Low instability<br>Medium instability<br>High instability<br>Most unstable<br>Missing	

Quintile of deprivation	CMG16_07_COF1<br>CMG16_07_TRF1	Least deprived<br>Low deprived<br>Medium deprived<br>High deprived<br>Most deprived<br>Missing	
Quintile of dependency	CMG16_08_COF1<br>CMG16_08_TRF1	Least dependent<br>Low dependent<br>Medium dependent<br>High dependent<br>Most dependent<br>Missing	
Quintile of ethnic concentration	CMG16_08_COF1<br>CMG16_08_TRF1	Least ethnically concentrated<br>Low ethnically concentrated<br>Medium ethnically concentrated<br>High ethnically concentrated<br>Most ethnically concentrated<br>Missing	

Table S2. Final tuned hyperparameters used to build random forest models.

	**Influenza vaccination in the past 12 months**			**Pneumococcal vaccination ever**		
	Objective 3	Sensitivity – correlation	Sensitivity – missing data	Objective 3	Sensitivity – correlation	Sensitivity – missing data
ntree[a]	1500	1500	1500	1500	1500	1500
nodesize[b]	8	8	8	8	8	8
mtry[c]	3	3	3	5	7	7
Mean AUROC[d]	0.645	0.642	0.641	0.747	0.745	0.749

[a] ntree: Number of decision trees

[b] nodesize: Minimum node size

[c] mtry: Number of variable splits

[d] AUROC: Area under the receiver operating characteristic curve

Table S3. Descriptive characteristics and self-reported influenza vaccination in the past 12 months of CLSA participants at follow up 1 (2015-2018) by immigrant status.

Characteristic	Influenza vaccination in the past 12 months					
	Total		Non-immigrant		Immigrant	
	N[a]	%[b] (95% CI[c])	N[a]	%[b] (95% CI[c])	N[a]	%[b] (95% CI[c])
**Overall**	**23214**		**18776**	**81.8 (80.8, 82.7)[i]**	**4438**	**18.2 (17.3, 19.2)[i]**
Influenza vaccination in the past 12 months						
No	6847	33.6 (32.4, 34.9)	5471	33.1 (31.7, 34.5)	1376	36.2 (33.3, 39.1)
Yes	16367	66.4 (65.1, 67.6)	13305	66.9 (65.5, 68.3)	3062	63.8 (60.9, 66.7)
Socio-demographic and -economic						
Age						
65-74	13192	62.4 (61.1, 63.6)	10806	62.6 (61.1, 63.9)	2386	61.5 (58.7, 64.3)
75-84	8259	30.9 (29.7, 32.1)	6565	30.8 (29.5, 32.1)	1694	31.6 (28.9, 34.3)
85+	1763	6.7 (6.2, 7.3)	1405	6.7 (6.1, 7.3)	358	6.9 (5.8, 8.3)
Sex at birth						
Female	11650	53.3 (51.9, 54.6)	9616	53.9 (52.5, 55.4)	2034	50.2 (47.2, 53.2)
Male	11564	46.7 (45.4, 48.1)	9160	46.1 (44.6, 47.5)	2404	49.8 (46.8, 52.8)
Sexual orientation						
Heterosexual	22636	97.6 (97.1, 97.9)	18299	97.5 (97.1, 97.9)	4337	97.8 (96.4, 98.6)
Homosexual	280	0.9 (0.7, 1.2)	235	1.0 (0.8, 1.3)	45	0.6 (0.4, 1.0)
Bisexual	133	0.6 (0.4, 0.8)	108	0.6 (0.5, 0.9)	25	0.3 (0.2, 0.8)
Other	67	0.2 (0.2, 0.4)	63	0.3 (0.2, 0.5)	4	<0.1 (0.0, 0.2)
Missing	98	0.7 (0.5, 1.0)	71	0.6 (0.4, 0.9)	27	1.2 (0.5, 2.8)
Current marital/partner status						
Married/common-law	14502	66.4 (65.2, 67.6)	11616	65.7 (64.3, 67.0)	2886	69.6 (66.9, 72.2)
Divorced/separated	2862	10.4 (9.7, 11.2)	2308	10.5 (9.7, 11.4)	554	10.1 (8.6, 11.8)
Single, never married/lived with a partner	1531	5.9 (5.4, 6.6)	1308	6.3 (5.6, 7.0)	223	4.4 (3.4, 5.6)
Widowed	4305	17.2 (16.2, 18.2)	3531	17.5 (16.4, 18.6)	774	15.9 (13.8, 18.2)
Missing	14	<0.1 (0.0, 0.1)	13	<0.1 (0.0, 0.1)	1	<0.1 (0.0, 0.2)
Race and ethnocultural background[d]						
White	22370	96.4 (95.8, 96.8)	18488	98.2 (97.8, 98.6)	3882	87.9 (85.9, 89.7)
East Asian	115	0.4 (0.3, 0.6)	41	0.2 (0.1, 0.3)	74	1.5 (1.0, 2.4)
Southeast Asian	51	0.2 (0.1, 0.3)	1	<0.1 (0.0, 0.1)	50	1.0 (0.6, 1.6)
South Asian	164	0.6 (0.4, 0.8)	0	0.0 (0.0, 0.0)	164	3.0 (2.2, 4.2)
Middle Eastern	28	<0.1 (0.0, 0.1)	4	<0.1 (0.0, 0.0)	24	0.4 (0.2, 0.6)
Black	116	0.7 (0.5, 1.1)	15	0.2 (0.1, 0.8)	101	2.9 (2.0, 4.1)
Other	345	1.5 (1.2, 1.9)	213	1.2 (1.0, 1.6)	132	2.8 (1.9, 4.2)
Missing	25	0.1 (0.1, 0.3)	14	<0.1 (0.0, 0.1)	11	0.4 (0.2, 1.3)
Highest level of education						
Post-secondary degree/diploma	16662	52.7 (51.4, 54.1)	13139	50.5 (49.0, 52.0)	3523	62.7 (59.4, 65.8)
Some post-secondary education	1851	10.5 (9.7, 11.3)	1522	10.5 (9.7, 11.3)	329	10.5 (8.8, 12.4)

Secondary school graduation	2641	15.8 (14.9, 16.8)	2261	16.2 (15.2, 17.3)	380	14.0 (12.0, 16.3)
Less than secondary school graduation	1987	20.7 (19.3, 22.1)	1806	22.6 (21.0, 24.2)	181	12.1 (9.5, 15.4)
Missing	73	0.4 (0.2, 0.6)	48	0.3 (0.2, 0.5)	25	0.7 (0.3, 1.6)
Total household income						
Less than $20,000	1299	6.9 (6.1, 7.6)	1108	7.4 (6.6, 8.4)	191	4.2 (3.2, 5.6)
$20,000 - <$50,000	7055	35.3 (34.0, 36.6)	5801	36.0 (34.6, 37.4)	1254	32.2 (29.4, 35.1)
$50,000 - <$100,000	8555	34.5 (33.2, 35.8)	6917	33.9 (32.5, 35.3)	1638	37.1 (34.2, 40.1)
$100,000 - <$150,000	2860	9.9 (9.2, 10.6)	2235	9.4 (8.7, 10.2)	625	11.9 (10.3, 13.8)
$150,000+	1450	4.7 (4.3, 5.2)	1091	4.5 (4.0, 5.0)	359	5.7 (4.7, 6.9)
Missing	1995	8.8 (8.0, 9.6)	1624	8.7 (8.0, 9.6)	371	8.9 (7.2, 10.9)
Religion[e]						
Christianity	17862	80.0 (79.0, 81.0)	15021	82.4 (81.3, 83.4)	2841	69.4 (66.7, 72.0)
Other	896	2.8 (2.5, 3.2)	519	2.1 (1.8, 2.5)	377	6.0 (4.8, 7.5)
None	4358	16.8 (15.8, 17.7)	3162	15.1 (14.1, 16.2)	1196	24.1 (21.8, 26.6)
Missing	98	0.4 (0.3, 0.6)	74	0.4 (0.3, 0.6)	24	0.5 (0.3, 0.9)
Language most spoken at home						
English	18620	73.7 (72.7, 74.6)	14735	71.4 (70.3, 72.4)	3885	83.9 (81.6, 86.1)
French	4224	24.4 (23.5, 25.3)	3998	28.4 (27.3, 29.4)	226	6.8 (5.4, 8.5)
Other	347	1.8 (1.5, 2.2)	28	0.2 (0.1, 0.4)	319	9.0 (7.4, 11.0)
Missing	23	0.1 (0.1, 0.2)	15	<0.1 (0.0, 0.2)	8	0.3 (0.1, 0.6)
Perceived socioeconomic standing						
Subjective financial situation						
Manage very well	11705	46.9 (45.6, 48.2)	9481	46.9 (45.5, 48.4)	2224	46.7 (43.8, 49.7)
Manage quite well	7372	33.2 (32.0, 34.5)	5928	33.0 (31.6, 34.4)	1444	34.3 (31.4, 37.3)
Get by alright	3333	15.5 (14.6, 16.5)	2711	15.6 (14.5, 16.7)	622	15.2 (13.2, 17.5)
Don't manage very well/have difficulties	536	3.0 (2.6, 3.5)	439	3.2 (2.6, 3.8)	97	2.4 (1.7, 3.5)
Missing	268	1.3 (1.1, 1.7)	217	1.3 (1.0, 1.7)	51	1.3 (0.8, 2.2)
Subjective standing in community						
Very high	2342	8.2 (7.6, 9.0)	1920	8.2 (7.4, 9.0)	422	8.6 (7.1, 10.4)
High	9418	35.2 (33.9, 36.4)	7636	35.6 (34.3, 37.0)	1782	33.1 (30.5, 35.8)
Medium	7951	38.9 (37.6, 40.3)	6459	38.8 (37.3, 40.2)	1492	39.8 (36.8, 42.9)
Low	1425	7.3 (6.7, 8.1)	1167	7.7 (6.9, 8.5)	258	5.7 (4.7, 7.0)
Very low	850	5.4 (4.8, 6.2)	686	5.5 (4.7, 6.3)	164	5.3 (3.9, 7.2)
Missing	1228	4.9 (4.4, 5.4)	908	4.3 (3.9, 4.9)	320	7.3 (6.0, 8.9)
Health status/access to healthcare						
Presence of chronic medical conditions						
No CMC	4951	21.8 (20.8, 22.9)	3947	21.6 (20.4, 22.8)	1004	23.0 (20.7, 25.5)
1+ CMC	17591	77.2 (76.1, 78.2)	14302	77.5 (76.3, 78.7)	3289	75.5 (73.0, 77.8)
Missing	672	1.0 (0.9, 1.2)	527	0.9 (0.8, 1.0)	145	1.5 (1.0, 2.2)

Functional impairment (ADL and IAL)						
No functional impairment	17987	76.8 (75.7, 77.9)	14512	76.4 (75.1, 77.7)	3475	78.6 (75.9, 81.1)
Some impairment	4535	20.6 (19.5, 21.7)	3704	20.9 (19.7, 22.2)	831	19.1 (16.7, 21.8)
Missing	692	2.6 (2.2, 3.0)	560	2.7 (2.3, 3.2)	132	2.3 (1.7, 2.9)
Unmet healthcare need in past 12 months						
No	21424	93.2 (92.5, 93.8)	17357	93.1 (92.4, 93.8)	4067	93.2 (91.8, 94.4)
Yes	1767	6.8 (6.1, 7.4)	1400	6.8 (6.1, 7.5)	367	6.8 (5.6, 8.1)
Missing	23	<0.1 (0.0, 0.2)	19	<0.1 (0.0, 0.2)	4	<0.1 (0.0, 0.2)
Contact with family doctor in past 12 months						
No	1277	6.2 (5.6, 6.9)	1022	6.3 (5.6, 7.1)	255	5.7 (4.6, 6.9)
Yes	21922	93.7 (93.1, 94.4)	17743	93.6 (92.8, 94.3)	4179	94.3 (93.1, 95.3)
Missing	15	<0.1 (0.0, 0.1)	11	<0.1 (0.0, 0.1)	4	<0.1 (0.0, 0.1)
Contact with medical specialist in past 12 months						
No	7455	34.8 (33.5, 36.1)	6071	35.0 (33.6, 36.4)	1384	33.8 (31.0, 36.8)
Yes	15715	65.1 (63.8, 66.3)	12668	64.8 (63.4, 66.2)	3047	66.0 (63.1, 68.9)
Missing	44	0.2 (0.1, 0.3)	37	0.2 (0.1, 0.3)	7	0.1 (0.1, 0.4)
Perceived health status						
Self-rated general health						
Excellent	3840	14.9 (14.0, 15.8)	3019	14.5 (13.5, 15.5)	821	16.6 (14.6, 18.8)
Very good	9275	38.3 (37.0, 39.6)	7598	38.8 (37.4, 40.2)	1677	36.2 (33.4, 39.1)
Good	7027	32.0 (30.8, 33.3)	5679	32.0 (30.7, 33.4)	1348	32.2 (29.4, 35.1)
Fair	2451	11.3 (10.5, 12.2)	1978	11.4 (10.4, 12.4)	473	11.1 (9.4, 13.2)
Poor	559	3.1 (2.6, 3.7)	463	3.1 (2.6, 3.7)	96	2.9 (1.8, 4.6)
Missing	62	0.4 (0.2, 0.7)	39	0.2 (0.1, 0.5)	23	0.9 (0.4, 2.5)
Self-rated mental health						
Excellent	5937	24.3 (23.2, 25.4)	4768	24.4 (23.1, 25.6)	1169	24.1 (21.7, 26.6)
Very good	9699	41.9 (40.6, 43.2)	7916	42.3 (40.8, 43.7)	1783	40.4 (37.5, 43.3)
Good	6275	27.9 (26.7, 29.2)	5047	27.8 (26.4, 29.1)	1228	28.7 (26.1, 31.6)
Fair	1120	4.8 (4.3, 5.4)	898	4.6 (4.1, 5.2)	222	5.7 (4.3, 7.5)
Poor	141	0.8 (0.6, 1.2)	118	0.8 (0.6, 1.1)	23	0.9 (0.3, 2.6)
Missing	42	0.2 (0.1, 0.4)	29	0.2 (0.1, 0.5)	13	0.3 (0.1, 0.7)
Self-rated healthy aging						
Excellent	4288	17.8 (16.8, 18.8)	3411	17.6 (16.5, 18.7)	877	18.9 (16.6, 21.4)
Very good	9949	42.1 (40.8, 43.4)	8109	42.4 (40.9, 43.8)	1840	40.8 (37.9, 43.8)
Good	6918	31.0 (29.8, 32.2)	5598	30.9 (29.5, 32.3)	1320	31.3 (28.6, 34.1)
Fair	1634	7.2 (6.6, 8.0)	1319	7.1 (6.4, 7.9)	315	7.6 (6.1, 9.6)
Poor	333	1.6 (1.3, 2.0)	272	1.7 (1.3, 2.2)	61	1.2 (0.7, 1.9)
Missing	92	0.3 (0.2, 0.5)	67	0.3 (0.2, 0.5)	25	0.2 (0.1, 0.4)
Lifestyle or health behaviour						

Smoking cigarettes						
Not at all	21999	93.5 (92.7, 94.1)	17760	93.2 (92.3, 93.9)	4239	94.8 (93.2, 96.0)
Occasionally	210	1.3 (1.0, 1.6)	173	1.2 (0.9, 1.5)	37	1.7 (0.9, 3.2)
Daily	978	5.2 (4.6, 5.9)	822	5.6 (4.9, 6.4)	156	3.5 (2.7, 4.6)
Missing	27	<0.1 (0.0, 0.1)	21	<0.1 (0.0, 0.1)	6	<0.1 (0.0, 0.2)
Drinking alcohol						
Did not drink in the last 12 months	3589	16.8 (15.8, 17.9)	3010	18.0 (16.8, 19.2)	579	11.6 (9.9, 13.5)
Occasional drinker	3212	14.8 (13.9, 15.8)	2657	15.0 (14.0, 16.2)	555	13.9 (11.8, 16.3)
Regular drinker	16371	68.2 (66.9, 69.5)	13073	66.8 (65.4, 68.2)	3298	74.5 (71.7, 77.0)
Missing	42	0.2 (0.1, 0.3)	36	0.2 (0.1, 0.3)	6	<0.1 (0.0, 0.2)
Satisfaction with life						
Satisfied/extremely satisfied	17291	73.8 (72.6, 74.9)	14098	74.2 (72.9, 75.5)	3193	71.7 (68.8, 74.3)
Neutral or slightly dis/satisfied	4590	20.3 (19.3, 21.5)	3669	20.4 (19.2, 21.6)	921	20.2 (17.9, 22.7)
Extremely dissatisfied/dissatisfied	855	3.8 (3.3, 4.4)	669	3.7 (3.1, 4.2)	186	4.5 (3.2, 6.2)
Missing	478	2.1 (1.8, 2.4)	340	1.7 (1.4, 2.1)	138	3.6 (2.6, 5.1)
Number of people living in household						
No other person living in household	7280	28.0 (26.9, 29.2)	5992	28.7 (27.4, 30.0)	1288	25.0 (22.7, 27.6)
1 other person living in household	13486	61.9 (60.6, 63.1)	10891	61.4 (59.9, 62.8)	2595	64.0 (61.2, 66.7)
2+ other persons living in household	2137	9.7 (8.9, 10.6)	1650	9.5 (8.6, 10.5)	487	10.3 (8.8, 12.1)
Missing	311	0.5 (0.4, 0.5)	243	0.4 (0.3, 0.5)	68	0.6 (0.4, 0.9)
Functional social support (MOS overall support index)[f]						
High social support	4591	19.3 (18.3, 20.4)	3740	19.4 (18.3, 20.6)	851	19.0 (16.8, 21.3)
Medium-high social support	6062	28.5 (27.4, 29.8)	4980	28.8 (24.1, 30.1)	1082	27.4 (24.9, 30.1)
Medium-low social support	5786	23.6 (22.5, 24.7)	4735	24.1 (22.9, 25.4)	1051	21.1 (18.9, 23.5)
Low social support	4949	20.2 (19.1, 21.2)	3910	20.0 (18.9, 21.2)	1039	20.7 (18.4, 23.2)
Missing	1826	8.4 (7.6, 9.2)	1411	7.6 (6.9, 8.5)	415	11.8 (9.5, 14.5)
Participation in providing assistance/caregiving in past 12 months						
Did not provide any assistance	11816	50.7 (49.4, 52.0)	9495	50.5 (49.0, 51.9)	2321	51.9 (48.9, 54.9)
Did provide assistance	11319	48.8 (47.4, 50.1)	9222	49.0 (47.6, 50.5)	2097	47.6 (44.6, 50.6)
Missing	79	0.5 (0.3, 0.8)	59	0.5 (0.3, 0.8)	20	0.5 (0.3, 1.1)
Participation in community-related activities in past 12 months						
Did not participate/participated once a year	693	4.7 (4.1, 5.4)	555	5.0 (4.3, 5.9)	138	3.4 (2.6, 4.4)
Participated at least once a month	2711	14.6 (13.6, 15.6)	2172	14.7 (13.5, 15.8)	539	14.2 (12.1, 16.5)
Participated at least once a week	15764	65.7 (64.4, 67.0)	12797	65.6 (64.1, 67.1)	2967	66.0 (63.0, 68.9)
Participated at least once a day	3692	14.5 (13.6, 15.5)	2974	14.2 (13.2, 15.3)	718	15.8 (13.5, 18.3)
Missing	354	0.5 (0.4, 0.6)	278	0.5 (0.4, 0.6)	76	0.7 (0.5, 1.0)
Environmental						
Province of residence						
Ontario	5165	33.6 (32.7, 34.6)	3776	31.3 (30.1, 32.5)	1389	44.3 (41.4, 47.2)

Newfoundland	1462	1.8 (1.7, 1.9)	1347	2.1 (1.9, 2.2)	115	0.5 (0.4, 0.7)
Prince Edward Island	507	0.4 (0.3, 0.4)	449	0.4 (0.4, 0.4)	58	0.2 (0.2, 0.3)
Nova Scotia	2096	3.2 (3.1, 3.4)	1801	3.5 (3.3, 3.7)	295	1.8 (1.5, 2.3)
New Brunswick	566	2.0 (1.8, 2.1)	523	2.2 (2.1, 2.4)	43	0.8 (0.6, 1.2)
Quebec	4316	26.6 (25.8, 27.4)	3920	29.4 (28.4, 30.4)	396	13.9 (12.0, 16.0)
Manitoba	2042	4.1 (3.9, 4.3)	1713	4.3 (4.1, 4.6)	329	2.9 (2.4, 3.4)
Saskatchewan	544	2.2 (2.0, 2.3)	504	2.5 (2.3, 2.7)	40	0.6 (0.4, 0.9)
Alberta	2243	8.6 (8.2, 8.9)	1764	8.6 (8.2, 9.1)	479	8.2 (7.0, 9.5)
British Columbia	4273	17.7 (17.1, 18.3)	2979	15.7 (15.0, 16.4)	1294	26.7 (24.6, 29.0)
Urbanicity of residence[g]						
Urban	19858	83.6 (82.6, 84.5)	15886	82.7 (81.6, 83.8)	3972	87.7 (85.6, 89.5)
Rural	3334	16.2 (15.3, 17.1)	2870	17.1 (16.0, 18.2)	464	12.3 (10.4, 14.4)
Missing	22	0.2 (0.1, 0.4)	20	0.2 (0.1, 0.5)	2	<0.1 (0.0, 0.2)
Quintile of instability[h]						
Least unstable	3322	15.8 (14.8, 16.8)	2573	15.4 (14.3, 16.5)	749	17.6 (15.5, 20.0)
Low instability	4250	18.9 (17.9, 20.0)	3434	18.6 (17.4, 19.8)	816	20.6 (18.1, 23.3)
Medium instability	4506	20.7 (19.7, 21.8)	3661	20.8 (19.7, 22.1)	845	20.3 (17.8, 23.1)
High instability	4862	20.0 (19.0, 21.0)	4035	20.4 (19.3, 21.6)	827	18.0 (16.0, 20.2)
Most unstable	6181	24.3 (23.2, 25.4)	4996	24.5 (23.3, 25.7)	1185	23.3 (21.1, 25.8)
Missing	93	0.3 (0.2, 0.4)	77	0.3 (0.2, 0.4)	16	0.2 (0.1, 0.4)
Quintile of deprivation[h]						
Least deprived	7476	26.3 (25.2, 27.3)	5652	24.8 (23.7, 26.0)	1824	32.8 (30.3, 35.5)
Low deprived	5123	21.0 (20.0, 22.2)	4100	20.3 (19.1, 21.5)	1023	24.4 (21.7, 27.3)
Medium deprived	4151	20.1 (19.0, 21.2)	3441	20.2 (19.0, 21.5)	710	19.7 (17.3, 22.4)
High deprived	3517	18.4 (17.3, 19.5)	2992	19.4 (18.2, 20.6)	525	13.9 (12.0, 16.2)
Most deprived	2854	13.9 (13.0, 14.9)	2514	15.0 (14.0, 16.1)	340	9.0 (7.6, 10.6)
Missing	93	0.3 (0.2, 0.4)	77	0.3 (0.2, 0.4)	16	0.2 (0.1, 0.4)
Quintile of dependency[h]						
Least dependent	2722	10.7 (9.9, 11.5)	2110	10.3 (9.5, 11.2)	612	12.2 (10.5, 14.0)
Low dependent	3683	15.9 (14.9, 16.9)	2937	15.6 (14.5, 16.7)	746	17.3 (15.2, 19.7)
Medium dependent	4527	19.6 (18.6, 20.7)	3638	19.2 (18.0, 20.4)	889	21.6 (19.2, 24.1)
High dependent	5048	22.2 (21.1, 23.4)	4098	22.4 (21.2, 23.7)	950	21.3 (18.9, 23.9)
Most dependent	7141	31.3 (30.1, 32.6)	5916	32.2 (30.9, 33.6)	1225	27.5 (24.8, 30.3)
Missing	93	0.3 (0.2, 0.4)	77	0.3 (0.2, 0.4)	16	0.2 (0.1, 0.4)
Quintile of ethnic concentration[h]						
Least ethnically concentrated	4444	22.0 (20.9, 23.1)	3810	23.4 (22.2, 24.7)	634	15.6 (13.4, 18.2)
Low ethnically concentrated	5474	21.7 (20.7, 22.8)	4589	22.6 (21.4, 23.8)	885	18.0 (15.9, 20.3)
Medium ethnically concentrated	5593	21.8 (20.7, 22.9)	4563	22.5 (21.3, 23.7)	1030	18.8 (16.6, 21.1)
High ethnically concentrated	4769	20.1 (19.1, 21.1)	3697	19.0 (17.9, 20.1)	1072	25.2 (22.8, 27.8)

Most ethnically concentrated	2841	14.1 (13.2, 15.1)	2040	12.3 (11.3, 13.3)	801	22.2 (19.8, 24.9)
Missing	93	0.3 (0.2, 0.4)	77	0.3 (0.2, 0.4)	16	0.2 (0.1, 0.4)

[a] N represents unweighted number of study participants
[b] % represents weighted proportions of study participants
[c] CI: Confidence intervals
[d] Categories of race and ethnocultural background were derived using the Canadian Institute for Health Information (CIHI) Guidance on the Use of Standards for Race-Based and Indigenous Identity Data Collection and Health Reporting in Canada (https://www.cihi.ca/sites/default/files/document/guidance-and-standards-for-race-based-and-indigenous-identity-data-en.pdf)
[e] Categories of religion were derived using the Statistics Canada Examples of religions and religious groups/denominations (https://www12.statcan.gc.ca/census-recensement/2021/ref/questionnaire/religion-eng.cfm)
[f] Functional social support categories were derived using quartiles of highest to lowest social support based on the results of the MOS Overall Support Index (https://www.clsa-elcv.ca/wp-content/uploads/2023/06/dv_ssa_11apr2018_v1.1.pdf)
[g] Urbanicity categories were defined by the Canadian Longitudinal Study on Aging (https://www.clsa-elcv.ca/wp-content/uploads/2023/06/urbanrural_dsd_01_03_2018_final.pdf)
[h] Dimensions of the Canadian Marginalization Index (https://www.ontariohealthprofiles.ca/onmarg/userguide_data/CAN-Marg_user_guide_1.0_FINAL_MAY2012.pdf)
[i] % represents the weighted proportions of non-immigrant and immigrant participants among total number of participants

Table S4. Descriptive characteristics and self-reported pneumococcal vaccination ever of CLSA participants at follow up 1 (2015-2018) by immigrant status.

Characteristic	Pneumococcal vaccination ever					
	Total		Non-immigrant		Immigrant	
	N[a]	%[b] (95% CI[c])	N[a]	%[b] (95% CI[c])	N[a]	%[b] (95% CI[c])
**Overall**	**22235**		**18013**	**81.9 (80.9, 82.9)[i]**	**4222**	**18.1 (17.1, 19.1)[i]**
Pneumococcal vaccination ever						
No	10185	45.5 (44.2, 46.9)	8034	44.2 (42.7, 45.7)	2151	51.3 (48.2, 54.4)
Yes	12050	54.5 (53.1, 55.8)	9979	55.8 (54.3, 57.3)	2071	48.7 (45.6, 51.8)
Socio-demographic and -economic						
Age						
65-74	12664	62.4 (61.1, 63.6)	10392	62.5 (61.1, 63.9)	2272	61.7 (58.8, 64.6)
75-84	7899	31.0 (29.8, 32.2)	6292	30.9 (29.6, 32.3)	1607	31.2 (28.5, 34.1)
85+	1672	6.7 (6.1, 7.3)	1329	6.6 (6.0, 7.2)	343	7.0 (5.8, 8.4)
Sex at birth						
Female	11362	54.0 (52.7, 55.4)	9401	54.8 (53.2, 56.2)	1961	50.7 (47.6, 53.8)
Male	10873	46.0 (44.6, 47.3)	8612	45.2 (43.8, 46.8)	2261	49.3 (46.2, 52.4)
Sexual orientation						
Heterosexual	21680	97.5 (97.1, 97.9)	17551	97.5 (97.0, 97.9)	4129	97.7 (96.2, 98.6)
Homosexual	268	0.9 (0.7, 1.1)	227	1.0 (0.7, 1.2)	41	0.6 (0.4, 1.0)
Bisexual	128	0.6 (0.4, 0.8)	105	0.7 (0.5, 0.9)	23	0.4 (0.2, 0.8)
Other	65	0.3 (0.2, 0.4)	62	0.3 (0.2, 0.5)	3	<0.1 (0.0, 0.2)
Missing	94	0.7 (0.5, 1.0)	68	0.6 (0.4, 0.9)	26	1.3 (0.5, 3.0)
Current marital/partner status						
Married/common-law	13874	66.5 (65.3, 67.8)	11132	65.9 (64.4, 67.3)	2742	69.6 (66.8, 72.2)
Divorced/separated	2729	10.3 (9.5, 11.1)	2198	10.3 (9.5, 11.2)	531	9.9 (8.5, 11.7)
Single, never married/lived with a partner	1473	5.9 (5.3, 6.6)	1261	6.3 (5.6, 7.0)	212	4.4 (3.4, 5.6)
Widowed	4146	17.2 (16.2, 18.3)	3410	17.5 (16.4, 18.6)	736	16.1 (14.0, 18.5)
Missing	13	<0.1 (0.0, 0.1)	12	<0.1 (0.0, 0.1)	1	<0.1 (0.0, 0.2)
Race and ethnocultural background[d]						
White	21428	96.4 (95.8, 96.8)	17735	98.2 (97.7, 98.6)	3693	87.9 (85.8, 89.8)
East Asian	109	0.4 (0.3, 0.6)	41	0.2 (0.1, 0.3)	68	1.6 (1.0, 2.4)
Southeast Asian	51	0.2 (0.1, 0.3)	1	<0.1 (0.0, 0.1)	50	1.1 (0.7, 1.7)
South Asian	156	0.6 (0.4, 0.8)	0	0.0 (0.0, 0.0)	156	3.1 (2.3, 4.3)
Middle Eastern	26	<0.1 (0.0, 0.1)	3	<0.1 (0.0, 0.0)	23	0.4 (0.2, 0.6)
Black	111	0.7 (0.5, 1.1)	15	0.3 (0.1, 0.8)	96	2.7 (1.9, 3.9)
Other	330	1.5 (1.2, 1.9)	204	1.2 (1.0, 1.6)	126	2.8 (1.8, 4.2)
Missing	24	0.1 (0.1, 0.3)	14	<0.1 (0.0, 0.1)	10	0.5 (0.2, 1.3)
Highest level of education						
Post-secondary degree/diploma	15973	52.7 (51.3, 54.0)	12619	50.5 (49.0, 52.0)	3354	62.5 (59.1, 65.7)

Some post-secondary education	1751	10.4 (9.6, 11.2)	1440	10.4 (9.6, 11.3)	311	10.4 (8.7, 12.4)
Secondary school graduation	2549	15.9 (14.9, 16.9)	2184	16.3 (15.2, 17.4)	365	14.0 (11.9, 16.4)
Less than secondary school graduation	1893	20.7 (19.3, 22.2)	1724	22.5 (20.9, 24.2)	169	12.5 (9.7, 15.9)
Missing	69	0.4 (0.2, 0.6)	46	0.3 (0.2, 0.5)	23	0.6 (0.3, 1.6)
Total household income						
Less than $20,000	1255	6.8 (6.1, 7.6)	1072	7.4 (6.6, 8.4)	183	4.2 (3.1, 5.5)
$20,000 - <$50,000	6787	35.3 (34.0, 36.6)	5591	36.0 (34.6, 37.5)	1196	32.0 (29.1, 35.0)
$50,000 - <$100,000	8198	34.6 (33.3, 35.9)	6640	34.0 (32.6, 35.4)	1558	37.1 (34.1, 40.2)
$100,000 - <$150,000	2727	9.9 (9.2, 10.7)	2136	9.4 (8.7, 10.3)	591	12.0 (10.4, 14.0)
$150,000+	1367	4.6 (4.1, 5.0)	1026	4.3 (3.8, 4.9)	341	5.7 (4.6, 6.9)
Missing	1901	8.8 (8.1, 9.6)	1548	8.8 (8.0, 9.7)	353	9.0 (7.3, 11.1)
Religion[e]						
Christianity	17131	80.1 (79.1, 81.1)	14423	82.4 (81.3, 83.5)	2708	69.6 (66.8, 72.3)
Other	847	2.8 (2.5, 3.2)	496	2.1 (1.8, 2.4)	351	6.1 (4.8, 7.7)
None	4162	16.6 (15.7, 17.6)	3022	15.0 (14.0, 16.1)	1140	23.8 (21.4, 26.3)
Missing	95	0.4 (0.3, 0.6)	72	0.4 (0.3, 0.7)	23	0.5 (0.3, 1.0)
Language most spoken at home						
English	17883	74.0 (73.1, 75.0)	14182	71.9 (70.7, 73.0)	3701	83.9 (81.4, 86.1)
French	4003	24.0 (23.1, 25.0)	3791	27.9 (26.8, 29.0)	212	6.7 (5.2, 8.4)
Other	327	1.8 (1.5, 2.2)	26	0.2 (0.1, 0.4)	301	9.2 (7.5, 11.2)
Missing	22	0.1 (0.1, 0.2)	14	<0.1 (0.0, 0.2)	8	0.3 (0.1, 0.6)
Perceived socioeconomic standing						
Subjective financial situation						
Manage very well	11206	47.0 (45.6, 48.3)	9097	47.1 (45.6, 48.6)	2109	46.3 (43.3, 49.4)
Manage quite well	7063	33.2 (31.9, 34.5)	5684	32.9 (31.5, 34.3)	1379	34.8 (31.9, 37.9)
Get by alright	3192	15.4 (14.4, 16.5)	2600	15.5 (14.4, 16.6)	592	15.2 (13.1, 17.5)
Don't manage very well/have difficulties	523	3.1 (2.6, 3.6)	429	3.2 (2.7, 3.9)	94	2.4 (1.7, 3.5)
Missing	251	1.3 (1.1, 1.7)	203	1.3 (1.0, 1.7)	48	1.2 (0.7, 2.2)
Subjective standing in community						
Very high	2222	8.2 (7.5, 9.0)	1833	8.2 (7.4, 9.0)	389	8.3 (6.8, 10.1)
High	9044	35.3 (34.1, 36.6)	7353	35.8 (34.4, 37.3)	1691	32.9 (30.2, 35.7)
Medium	7638	39.1 (37.7, 40.5)	6204	38.8 (37.3, 40.3)	1434	40.3 (37.2, 43.5)
Low	1363	7.2 (6.5, 8.0)	1112	7.5 (6.7, 8.4)	251	5.8 (4.8, 7.1)
Very low	821	5.5 (4.8, 6.3)	661	5.5 (4.8, 6.3)	160	5.6 (4.1, 7.5)
Missing	1147	4.7 (4.2, 5.2)	850	4.1 (3.7, 4.7)	297	7.1 (5.8, 8.7)
Health status/access to healthcare						
Presence of chronic medical conditions						
No CMC	4690	21.5 (20.4, 22.6)	3737	21.2 (20.0, 22.5)	953	22.7 (20.4, 25.2)
1+ CMC	16910	77.5 (76.4, 78.6)	13777	77.9 (76.6, 79.1)	3133	75.8 (73.3, 78.2)

Missing	635	1.0 (0.8, 1.1)	499	0.9 (0.8, 1.0)	136	1.4 (1.0, 2.1)
Functional impairment (ADL and IAL)						
No functional impairment	17220	76.8 (75.6, 78.0)	13914	76.5 (75.1, 77.7)	3306	78.4 (75.6, 81.0)
Some impairment	4346	20.6 (19.5, 21.7)	3553	20.8 (19.6, 22.1)	793	19.4 (16.8, 22.2)
Missing	669	2.6 (2.2, 3.1)	546	2.7 (2.3, 3.2)	123	2.2 (1.7, 2.9)
Unmet healthcare need in past 12 months						
No	20507	93.2 (92.5, 93.8)	16636	93.2 (92.4, 93.9)	3871	93.1 (91.7, 94.4)
Yes	1706	6.8 (6.1, 7.5)	1359	6.8 (6.0, 7.5)	347	6.8 (5.6, 8.3)
Missing	22	<0.1 (0.0, 0.2)	18	<0.1 (0.0, 0.2)	4	<0.1 (0.0, 0.2)
Contact with family doctor in past 12 months						
No	1216	6.1 (5.5, 6.8)	975	6.2 (5.5, 7.0)	241	5.6 (4.6, 6.9)
Yes	21007	93.9 (93.2, 94.5)	17030	93.8 (92.9, 94.5)	3977	94.3 (93.1, 95.4)
Missing	12	<0.1 (0.0, 0.1)	8	<0.1 (0.0, 0.1)	4	<0.1 (0.0, 0.1)
Contact with medical specialist in past 12 months						
No	7155	34.8 (33.5, 36.1)	5842	35.0 (33.6, 36.4)	1313	33.9 (30.9, 36.9)
Yes	15045	65.1 (63.8, 66.4)	12143	64.9 (63.4, 66.3)	2902	66.0 (62.9, 68.9)
Missing	35	0.1 (0.1, 0.2)	28	0.1 (0.1, 0.3)	7	0.2 (0.1, 0.4)
Perceived health status						
Self-rated general health						
Excellent	3697	15.0 (14.1, 15.9)	2916	14.6 (13.6, 15.7)	781	16.6 (14.5, 18.8)
Very good	8869	38.3 (37.0, 39.7)	7274	38.8 (37.3, 40.2)	1595	36.5 (33.6, 39.5)
Good	6731	32.0 (30.8, 33.3)	5445	32.0 (30.6, 33.5)	1286	32.0 (29.1, 34.9)
Fair	2344	11.2 (10.4, 12.2)	1898	11.3 (10.3, 12.3)	446	11.1 (9.3, 13.2)
Poor	536	3.0 (2.5, 3.6)	442	3.1 (2.5, 3.7)	94	3.0 (1.9, 4.8)
Missing	58	0.4 (0.2, 0.7)	38	0.2 (0.1, 0.5)	20	0.8 (0.3, 2.5)
Self-rated mental health						
Excellent	5706	24.3 (23.2, 25.5)	4586	24.3 (23.1, 25.6)	1120	24.2 (21.7, 26.8)
Very good	9293	42.1 (40.7, 43.4)	7605	42.5 (41.0, 44.0)	1688	40.0 (37.1, 43.1)
Good	5995	27.8 (26.6, 29.1)	4826	27.6 (26.2, 29.0)	1169	28.8 (26.0, 31.7)
Fair	1067	4.8 (4.2, 5.4)	856	4.5 (4.0, 5.1)	211	5.8 (4.4, 7.8)
Poor	136	0.8 (0.6, 1.2)	113	0.8 (0.6, 1.1)	23	0.9 (0.3, 2.7)
Missing	38	0.2 (0.1, 0.4)	27	0.2 (0.1, 0.5)	11	0.3 (0.1, 0.8)
Self-rated healthy aging						
Excellent	4119	17.9 (16.8, 18.9)	3287	17.6 (16.5, 18.8)	832	18.9 (16.5, 21.5)
Very good	9521	41.9 (40.6, 43.2)	7769	42.1 (40.7, 43.6)	1752	40.7 (37.7, 43.8)
Good	6629	31.1 (29.9, 32.4)	5369	31.1 (29.7, 32.5)	1260	31.4 (28.6, 34.3)
Fair	1557	7.2 (6.5, 7.9)	1262	7.1 (6.4, 7.9)	295	7.6 (6.0, 9.7)
Poor	319	1.6 (1.3, 2.0)	260	1.7 (1.3, 2.2)	59	1.2 (0.7, 2.0)
Missing	90	0.3 (0.2, 0.5)	66	0.3 (0.2, 0.6)	24	0.2 (0.1, 0.4)

Lifestyle or health behaviour						
Smoking cigarettes						
Not at all	21080	93.5 (92.8, 94.2)	17046	93.2 (92.4, 94.0)	4034	94.9 (93.2, 96.1)
Occasionally	202	1.3 (1.0, 1.7)	167	1.2 (0.9, 1.6)	35	1.7 (0.9, 3.3)
Daily	928	5.1 (4.5, 5.8)	780	5.5 (4.8, 6.3)	148	3.4 (2.6, 4.4)
Missing	25	<0.1 (0.0, 0.1)	20	<0.1 (0.0, 0.1)	5	<0.1 (0.0, 0.2)
Drinking alcohol						
Did not drink in the last 12 months	3445	16.9 (15.8, 18.0)	2889	18.0 (16.8, 19.3)	556	11.8 (10.1, 13.8)
Occasional drinker	3088	14.8 (13.8, 15.8)	2562	15.0 (13.9, 16.2)	526	13.9 (11.7, 16.3)
Regular drinker	15663	68.2 (66.8, 69.5)	12530	66.8 (65.3, 68.3)	3133	74.2 (71.4, 76.8)
Missing	39	0.2 (0.1, 0.3)	32	0.2 (0.1, 0.3)	7	0.1 (0.0, 0.3)
Flu vaccination in the past 12 months						
No	6574	33.6 (32.3, 34.9)	5254	33.0 (31.6, 34.5)	1320	36.2 (33.3, 39.3)
Yes	15631	66.3 (65.0, 67.6)	12734	66.9 (65.4, 68.3)	2897	63.7 (60.6, 66.6)
Missing	30	0.1 (0.1, 0.3)	25	0.1 (0.1, 0.4)	5	<0.1 (0.0, 0.3)
Satisfaction with life						
Satisfied/extremely satisfied	16585	73.9 (72.7, 75.1)	13541	74.4 (73.0, 75.7)	3044	71.9 (69.0, 74.6)
Neutral or slightly dis/satisfied	4379	20.3 (19.2, 21.4)	3498	20.2 (19.0, 21.5)	881	20.4 (18.1, 23.0)
Extremely dissatisfied/dissatisfied	829	3.8 (3.3, 4.4)	654	3.7 (3.2, 4.3)	175	4.4 (3.2, 6.2)
Missing	442	2.0 (1.6, 2.3)	320	1.7 (1.4, 2.0)	122	3.2 (2.2, 4.6)
Social support and activities						
Number of people living in household						
No other person living in household	6965	27.8 (26.6, 29.0)	5743	28.4 (27.1, 29.7)	1222	24.9 (22.4, 27.5)
1 other person living in household	12940	62.0 (60.7, 63.3)	10474	61.6 (60.1, 63.0)	2466	64.0 (61.1, 66.8)
2+ other persons living in household	2031	9.7 (8.9, 10.6)	1563	9.6 (8.6, 10.6)	468	10.6 (9.0, 12.4)
Missing	299	0.5 (0.4, 0.5)	233	0.4 (0.3, 0.5)	66	0.6 (0.4, 0.9)
Functional social support (MOS overall support index)[f]						
High social support	4408	19.4 (18.3, 20.4)	3602	19.5 (18.4, 20.7)	806	18.6 (16.4, 21.1)
Medium-high social support	5854	28.8 (27.6, 30.0)	4820	29.0 (27.7, 30.4)	1034	27.8 (25.2, 30.6)
Medium-low social support	5537	23.6 (22.4, 24.7)	4533	24.1 (22.8, 25.4)	1004	21.2 (18.9, 23.7)
Low social support	4717	20.1 (19.0, 21.1)	3731	19.9 (18.7, 21.1)	986	20.7 (18.4, 23.3)
Missing	1719	8.2 (7.4, 9.1)	1327	7.4 (6.7, 8.3)	392	11.6 (9.3, 14.5)
Participation in providing assistance/caregiving in past 12 months						
Did not provide any assistance	11311	50.8 (49.5, 52.2)	9100	50.5 (49.0, 52.0)	2211	52.1 (49.0, 55.1)
Did provide assistance	10850	48.7 (47.3, 50.0)	8857	49.0 (47.5, 50.5)	1993	47.4 (44.3, 50.5)
Missing	74	0.5 (0.3, 0.8)	56	0.5 (0.3, 0.8)	18	0.5 (0.2, 1.1)
Participation in community-related activities in past 12 months						
Did not participate/participated once a year	657	4.5 (3.9, 5.2)	523	4.8 (4.1, 5.6)	134	3.4 (2.6, 4.5)
Participated at least once a month	2579	14.5 (13.5, 15.6)	2068	14.6 (13.5, 15.8)	511	14.3 (12.2, 16.7)

Participated at least once a week	15130	65.9 (64.6, 67.2)	12302	65.9 (64.4, 67.3)	2828	66.2 (63.1, 69.1)
Participated at least once a day	3530	14.5 (13.5, 15.5)	2855	14.3 (13.3, 15.4)	675	15.4 (13.1, 18.0)
Missing	339	0.5 (0.4, 0.6)	265	0.5 (0.4, 0.6)	74	0.7 (0.5, 1.0)
Environmental						
Province of residence						
Ontario	4913	33.8 (32.8, 34.7)	3601	31.4 (30.2, 32.7)	1312	44.3 (41.3, 47.3)
Newfoundland	1430	1.8 (1.7, 1.9)	1318	2.1 (2.0, 2.2)	112	0.5 (0.4, 0.7)
Prince Edward Island	490	0.4 (0.3, 0.4)	436	0.4 (0.4, 0.4)	54	0.2 (0.2, 0.3)
Nova Scotia	1976	3.2 (3.0, 3.3)	1705	3.5 (3.3, 3.7)	271	1.8 (1.4, 2.2)
New Brunswick	534	1.9 (1.8, 2.0)	493	2.2 (2.0, 2.3)	41	0.8 (0.6, 1.2)
Quebec	4113	26.3 (25.5, 27.1)	3737	29.0 (28.0, 30.1)	376	13.9 (12.0, 16.1)
Manitoba	1948	4.0 (3.8, 4.2)	1629	4.3 (4.0, 4.5)	319	2.9 (2.5, 3.5)
Saskatchewan	532	2.2 (2.1, 2.3)	491	2.5 (2.4, 2.7)	41	0.7 (0.5, 1.0)
Alberta	2186	8.7 (8.3, 9.1)	1726	8.8 (8.3, 9.3)	460	8.2 (7.0, 9.5)
British Columbia	4113	17.8 (17.2, 18.4)	2877	15.8 (15.0, 16.5)	1236	26.7 (24.5, 29.0)
Urbanicity of residence[g]						
Urban	19011	83.6 (82.6, 84.5)	15232	82.7 (81.6, 83.8)	3779	87.5 (85.3, 89.4)
Rural	3203	16.2 (15.3, 17.2)	2761	17.1 (16.0, 18.2)	442	12.5 (10.6, 14.7)
Missing	21	0.2 (0.1, 0.4)	20	0.2 (0.1, 0.5)	1	<0.1 (0.0, 0.3)
Quintile of instability[h]						
Least unstable	3174	15.9 (14.9, 16.9)	2462	15.5 (14.4, 16.7)	712	17.6 (15.4, 20.0)
Low instability	4066	19.0 (17.9, 20.1)	3297	18.7 (17.5, 20.0)	769	20.3 (17.8, 23.1)
Medium instability	4329	20.8 (19.7, 21.9)	3522	20.8 (19.6, 22.1)	807	20.4 (17.8, 23.3)
High instability	4668	19.9 (18.9, 21.0)	3874	20.3 (19.2, 21.5)	794	18.3 (16.2, 20.6)
Most unstable	5907	24.1 (23.0, 25.2)	4783	24.3 (23.1, 25.6)	1124	23.2 (20.9, 25.7)
Missing	91	0.3 (0.2, 0.4)	75	0.3 (0.2, 0.4)	16	0.2 (0.1, 0.4)
Quintile of deprivation[h]						
Least deprived	7149	26.3 (25.2, 27.4)	5404	24.8 (23.7, 26.1)	1745	32.9 (30.2, 35.6)
Low deprived	4889	21.0 (19.9, 22.1)	3930	20.3 (19.1, 21.6)	959	23.9 (21.2, 27.0)
Medium deprived	4003	20.2 (19.1, 21.4)	3318	20.3 (19.1, 21.6)	685	20.0 (17.5, 22.8)
High deprived	3373	18.3 (17.2, 19.4)	2876	19.2 (18.0, 20.5)	497	14.0 (12.0, 16.3)
Most deprived	2730	13.9 (13.0, 14.9)	2410	15.0 (13.9, 16.1)	320	9.0 (7.6, 10.7)
Missing	91	0.3 (0.2, 0.4)	75	0.3 (0.2, 0.4)	16	0.2 (0.1, 0.4)
Quintile of dependency[h]						
Least dependent	2597	10.6 (9.9, 11.4)	2015	10.3 (9.5, 11.2)	582	12.0 (10.3, 13.9)
Low dependent	3531	15.9 (15.0, 17.0)	2819	15.6 (14.5, 16.8)	712	17.4 (15.2, 19.9)
Medium dependent	4323	19.6 (18.5, 20.7)	3482	19.1 (18.0, 20.4)	841	21.6 (19.1, 24.2)
High dependent	4838	22.1 (21.0, 23.3)	3943	22.4 (21.1, 23.7)	895	20.9 (18.5, 23.6)
Most dependent	6855	31.5 (30.2, 32.7)	5679	32.2 (30.9, 33.6)	1176	27.9 (25.2, 30.9)

Missing	91	0.3 (0.2, 0.4)	75	0.3 (0.2, 0.4)	16	0.2 (0.1, 0.4)
Quintile of ethnic concentration[h]						
Least ethnically concentrated	4267	22.0 (20.9, 23.2)	3666	23.4 (22.1, 24.7)	601	15.9 (13.5, 18.5)
Low ethnically concentrated	5267	21.9 (20.8, 23.0)	4423	22.7 (21.5, 23.9)	844	18.2 (16.0, 20.6)
Medium ethnically concentrated	5341	21.8 (20.7, 22.9)	4357	22.5 (21.2, 23.8)	984	18.6 (16.5, 21.0)
High ethnically concentrated	4542	19.9 (18.9, 21.0)	3527	18.8 (17.8, 20.0)	1015	24.7 (22.2, 27.4)
Most ethnically concentrated	2727	14.2 (13.2, 15.1)	1965	12.3 (11.4, 13.4)	762	22.4 (19.9, 25.1)
Missing	91	0.3 (0.2, 0.4)	75	0.3 (0.2, 0.4)	16	0.2 (0.1, 0.4)

[a] N represents unweighted number of study participants

[b] % represents weighted proportions of study participants

[c] CI: Confidence intervals

[d] Categories of race and ethnocultural background were derived using the Canadian Institute for Health Information (CIHI) Guidance on the Use of Standards for Race-Based and Indigenous Identity Data Collection and Health Reporting in Canada (https://www.cihi.ca/sites/default/files/document/guidance-and-standards-for-race-based-and-indigenous-identity-data-en.pdf)

[e] Categories of religion were derived using the Statistics Canada Examples of religions and religious groups/denominations (https://www12.statcan.gc.ca/census-recensement/2021/ref/questionnaire/religion-eng.cfm)

[f] Functional social support categories were derived using quartiles of highest to lowest social support based on the results of the MOS Overall Support Index (https://www.clsa-elcv.ca/wp-content/uploads/2023/06/dv_ssa_11apr2018_v1.1.pdf)

[g] Urbanicity categories were defined by the Canadian Longitudinal Study on Aging (https://www.clsa-elcv.ca/wp-content/uploads/2023/06/urbanrural_dsd_01_03_2018_final.pdf)

[h] Dimensions of the Canadian Marginalization Index (https://www.ontariohealthprofiles.ca/onmarg/userguide_data/CAN-Marg_user_guide_1.0_FINAL_MAY2012.pdf)

[i] % represents the weighted proportions of non-immigrant and immigrant participants among total number of participants

Table S5. Performance measures of random forest models from primary and sensitivity analyses using test data set.

	**Influenza vaccination in the past 12 months**			**Pneumococcal vaccination ever**		
	Objective 3	Sensitivity – correlation	Sensitivity – missing data	Objective 3	Sensitivity – correlation	Sensitivity – missing data
Sensitivity	0.97	0.98	0.97	0.83	0.84	0.81
Specificity	0.09	0.07	0.11	0.59	0.59	0.59
PPV[a]	0.72	0.72	0.73	0.69	0.70	0.69
NPV[b]	0.60	0.66	0.58	0.75	0.77	0.73
AUROC[c]	0.66	0.65	0.66	0.77	0.77	0.75

[a] PPV: Positive predictive value

[b] NPV: Negative predictive value

[c] AUROC: Area under the receiver operating characteristic curve

Table S6. Prevalence of influenza and pneumococcal vaccination in CLSA participants at follow up 1 (2015-2018) using imputed data.

	**Influenza vaccination in the past 12 months**		**Pneumococcal vaccination ever**	
	PR[a] (95% CI[b])	aPR[c] (95% CI[b])	PR[a] (95% CI[b])	aPR[c] (95% CI[b])
Immigrant status				
Non-immigrant	Ref	Ref	Ref	Ref
Immigrant	0.96 (0.93, 0.99)	0.93 (0.90, 0.96)	0.89 (0.85, 0.93)	0.88 (0.84, 0.92)
Age				
65-74	Ref	Ref	Ref	Ref
75-84	1.20 (1.17, 1.22)	1.21 (1.19, 1.24)	1.31 (1.26, 1.35)	1.32 (1.28, 1.36)
85 and over	1.24 (1.19, 1.29)	1.27 (1.22, 1.31)	1.34 (1.27, 1.41)	1.35 (1.28, 1.43)
Sex at birth				
Female	Ref	Ref	Ref	Ref
Male	1.01 (0.98, 1.03)	0.99 (0.97, 1.02)	0.88 (0.85, 0.91)	0.87 (0.85, 0.90)
Race and ethnocultural background[d]				
White	Ref	Ref	Ref	Ref
Racialized	0.90 (0.84, 0.97)	0.94 (0.88, 1.01)	0.85 (0.77, 0.94)	0.92 (0.83, 1.02)
Highest level of education				
Post-secondary degree/diploma	Ref	Ref	Ref	Ref
Some post-secondary education	0.98 (0.94, 1.02)	0.97 (0.93, 1.00)	1.00 (0.95, 1.05)	0.97 (0.92, 1.03)
Secondary school graduation	0.97 (0.94, 1.00)	0.97 (0.94, 1.00)	1.01 (0.97, 1.06)	0.99 (0.94, 1.03)
Less than secondary school graduation	0.97 (0.93, 1.00)	0.96 (0.93, 1.00)	1.04 (0.99, 1.09)	0.98 (0.93, 1.03)
Total household income				
Less than $20,000	Ref	Ref	Ref	Ref
$20,000 - <$50,000	1.12 (1.05, 1.18)	1.09 (1.03, 1.16)	1.00 (0.94, 1.08)	1.05 (0.98, 1.12)
$50,000 - <$100,000	1.20 (1.13, 1.27)	1.18 (1.11, 1.25)	1.05 (0.98, 1.13)	1.15 (1.07, 1.24)
$100,000 - <$150,000	1.26 (1.18, 1.34)	1.24 (1.16, 1.32)	1.08 (1.00, 1.17)	1.21 (1.12, 1.31)
$150,000+	1.21 (1.13, 1.30)	1.19 (1.11, 1.28)	1.00 (0.92, 1.10)	1.13 (1.03, 1.24)
Province of residence				
Ontario	Ref	Ref	Ref	Ref
Newfoundland	0.86 (0.81, 0.90)	0.86 (0.82, 0.91)	0.59 (0.54, 0.65)	0.59 (0.54, 0.65)
Prince Edward Island	0.97 (0.90, 1.05)	1.00 (0.93, 1.08)	0.79 (0.69, 0.90)	0.80 (0.70, 0.92)
Nova Scotia	1.10 (1.06, 1.14)	1.11 (1.08, 1.15)	0.96 (0.90, 1.03)	0.97 (0.91, 1.03)
New Brunswick	0.94 (0.87, 1.01)	0.98 (0.91, 1.05)	0.79 (0.70, 0.90)	0.81 (0.71, 0.92)
Quebec	0.77 (0.74, 0.80)	0.79 (0.76, 0.82)	1.08 (1.03, 1.13)	1.09 (1.04, 1.15)
Manitoba	0.93 (0.89, 0.98)	0.94 (0.90, 0.98)	1.12 (1.05, 1.19)	1.12 (1.05, 1.19)
Saskatchewan	0.92 (0.86, 0.99)	0.92 (0.86, 1.00)	1.03 (0.93, 1.14)	1.02 (0.92, 1.12)
Alberta	0.96 (0.92, 1.00)	0.96 (0.92, 1.00)	1.13 (1.07, 1.20)	1.14 (1.08, 1.20)
British Columbia	0.92 (0.89, 0.95)	0.92 (0.89, 0.95)	0.98 (0.93, 1.03)	0.99 (0.94, 1.04)
Urbanicity of residence[e]				
Urban	Ref	Ref	Ref	Ref
Rural	0.94 (0.91, 0.97)	0.95 (0.91, 0.98)	0.91 (0.86, 0.95)	0.97 (0.92, 1.02)

[a] PR: prevalence ratio
[b] CI: confidence interval
[c] aPR: adjusted prevalence ratio
[d] Categories of race and ethnocultural background were derived using the Canadian Institute for Health Information (CIHI) Guidance on the Use of Standards for Race-Based and Indigenous Identity Data Collection and Health Reporting in Canada (https://www.cihi.ca/sites/default/files/document/guidance-and-standards-for-race-based-and-indigenous-identity-data-en.pdf)
[e] Urbanicity categories were defined by the Canadian Longitudinal Study on Aging (https://www.clsa-elcv.ca/wp-content/uploads/2023/06/urbanrural_dsd_01_03_2018_final.pdf)
